# Brain alterations detected by multiplex magnetic resonance imaging in nasopharyngeal carcinoma patients after induction chemotherapy: a longitudinal study

**DOI:** 10.7717/peerj.21650

**Published:** 2026-08-14

**Authors:** Dan Liu, Kai Yang, Panpan Dong, Zien Chen, Shuangquan Ai, Xiaopeng Song, Tao You, Jianping Bi, Zilong Yuan, Xiaofang Guo, Yulin Liu

**Affiliations:** 1 Department of Radiology, Hubei Cancer Hospital, Tongji Medical College, Huazhong University of Science and Technology, Wuhan, China; 2 School of Biomedical Engineering, South-Central Minzu University, Wuhan, China; 3 Central Research Institute, United Imaging Healthcare, Shanghai, China; 4 Department of Radiology, Chinese PLA General Hospital of Central Theater Command, Wuhan, China; 5 Department of Radiation Oncology, Hubei Cancer Hospital, Tongji Medical College, Huazhong University of Science and Technology, Wuhan, China

**Keywords:** Nasopharyngeal carcinoma (NPC), Multiplex (MTP) MR imaging, Induction chemotherapy (ICT), Brain region quantitative properties, Cognitive decline

## Abstract

**Background:**

Alterations in brain regions and cognitive changes in patients with nasopharyngeal carcinoma (NPC) after induction chemotherapy (ICT) remain overlooked. This study aimed to investigate brain alterations and their cognitive correlations using a novel Multiplex (MTP) magnetic resonance imaging (MRI) sequence.

**Methods:**

Thirty-nine patients underwent MTP imaging and Montreal Cognitive Assessment (MoCA) test before (pre-ICT) and after (post-ICT) ICT. Paired-samples permutation test determined the differences in quantitative MRI parameters of brain regions between pre- and post-ICT assessments. Multiple linear regression analysis, adjusted for age, sex, and education, examined the relationship between longitudinal changes in regional parameters and MoCA scores. Analysis of covariance, controlling for baseline MoCA scores and demographic variables, compared the parameters of longitudinally altered brain regions between MoCA-decreased (MoCA-dec) and MoCA-non-decreased (MoCA-nd) groups. Multivariate logistic regression and receiver operating characteristic curve analysis evaluated the combined performance of clinical variables and brain regional parameters in predicting cognitive impairment.

**Results:**

Post-ICT patients exhibited decreased volume and R2*, as well as increased T2* and quantitative susceptibility mapping values, predominantly in the default mode network, occipital lobe, temporal lobe, and subcortical regions. Baseline R2* values in the right superior frontal gyrus, left cuneus, and left lingual gyrus showed significant false discovery rate (FDR)-corrected negative associations with post-ICT naming subscores. The MoCA-dec group exhibited significantly larger right amygdala volumes both before and after ICT, and smaller left inferior temporal gyrus (ITG.L) volumes after ICT compared with the MoCA-nd group. A combined model of sex, baseline MoCA score, and post-ICT ITG.L volume achieved a preliminary and exploratory area under the curve (AUC) of 0.706 for predicting cognitive decline.

**Conclusions:**

MTP demonstrates potential for evaluating brain alterations in NPC following ICT, and these multiparametric indices showed preliminary associations with cognitive decline. However, these findings should be interpreted as exploratory, as they were derived from a cohort without a non-ICT control group. In addition, the single post-ICT time point cannot determine whether the observed alterations are transient, persistent, or progressive. Consequently, this exploratory study provides neurobiological insights into ICT-related cognitive changes and generates hypotheses regarding the role of MTP-derived parameters as imaging biomarkers in future studies.

## Introduction

Nasopharyngeal carcinoma (NPC) is a prevalent malignancy of the head and neck. Approximately 75% of newly diagnosed NPC cases in China are locally advanced NPC (LANPC) (Ho et al., 2012). Although radiotherapy is the primary treatment, the 5-year overall survival (OS) for LANPC remains suboptimal, ranging from 41% to 63%. The addition of concurrent chemotherapy resulted in only a modest (4%–6%) improvement in 5-year OS. However, distant metastasis remains the main cause of treatment failure. Therefore, it is imperative to improve the clinical outcomes of LANPC. Previous studies have demonstrated that the inclusion of induction chemotherapy (ICT) in concurrent chemoradiotherapy (CCRT) leads to reduced distant failure (Chen et al., 2018b). It also improved distant metastasis-free survival (Tang et al., 2020; Wang et al., 2020) (91.5% *versus* 79.4%), progression-free survival (Tang et al., 2020), and OS (Chen et al., 2018b) compared to patients with LANPC treated with CCRT alone. Consequently, pre-irradiation ICT has become the standard therapeutic option for patients with LANPC (Wu et al., 2022; Zhang et al., 2022b). Despite its clinical efficacy, chemotherapy may release neurotoxic agents that disrupt the blood–brain barrier, alter neuroinflammatory responses, increase oxidative stress, and cause mitochondrial dysfunction (Wefel & Schagen, 2012; Mounier et al., 2020). By compromising the integrity of white matter (Matsos et al., 2017) and gray matter (Niu et al., 2021) in the central nervous system, these factors drive the development of chemotherapy-induced brain changes. These alterations may contribute to chemotherapy-related cognitive impairment (CRCI) (Wefel & Schagen, 2012; Schagen et al., 2022), thereby impairing the patient’s quality of life. However, current studies are limited in their ability to visualize or quantify chemotherapy-induced brain changes.

Numerous studies have established that radiation therapy induces brain changes in patients with NPC, as demonstrated by various neuroimaging techniques (Leng et al., 2017; Yang et al., 2019; Kang et al., 2022; Zhang et al., 2022a; Zhang et al., 2020a). However, fewer studies have directly compared the differential effects of chemotherapy and radiotherapy on brain structure and function. Recent longitudinal studies have indicated distinct temporal patterns: chemotherapy may induce transient compensatory increases in regional brain activity, whereas radiotherapy results in persistent functional alterations (Wu et al., 2025). Structural magnetic resonance imaging (MRI) studies have demonstrated that radiotherapy induces loss of white matter integrity and gray matter atrophy, primarily within the radiation field (Leng et al., 2017; Yang et al., 2019; Zhang et al., 2020a). Conversely, chemotherapy-related brain changes are more diffuse, affecting default mode network (DMN) regions and subcortical structures. Some evidence suggests that these changes may precede or occur independently of radiation effects (Zhang et al., 2019b). The relative contributions of chemotherapy and radiotherapy to cognitive impairment in patients with NPC remain unclear, particularly when treatments are administered sequentially or concurrently, making it difficult to isolate chemotherapy-specific effects. Consequently, few NPC studies have successfully isolated the effects of chemotherapy alone. However, the evidence from other malignancies, such as breast cancer (BC), demonstrates that chemotherapy is associated with brain alterations and cognitive decline (Tong et al., 2020; Zhou et al., 2022; Yang et al., 2023). This reinforces the plausibility that similar mechanisms may exist in NPC and require dedicated studies. Because ICT is the standard treatment for NPC, inclusion of a non-ICT control group was not ethically feasible. Therefore, the present study employed a within-subject longitudinal design to characterize ICT-related brain alterations during the ICT phase rather than establish causality.

MRI-based indices have been established as neuroimaging biomarkers for evaluating post-treatment cognitive impairment in cancer patients. For instance, resting-state functional MRI (rs-fMRI) metrics, such as regional homogeneity (ReHo) and fractional amplitude of low-frequency fluctuations, are sensitive biomarkers. These markers are used to monitor treatment-related brain changes and their correlation with cognitive decline in patients with NPC following CCRT (Wu et al., 2025). Similarly, MRI-derived brain region volume is a validated neuroimaging biomarker for the early detection of post-radiotherapy cognitive decline in patients with NPC, as volumetric alterations are consistently associated with cognitive impairment in patients with radiation-induced brain injury (Voon, Manan & Yahya, 2024). Furthermore, degree centrality and morphological network analyses have demonstrated that global efficiency and characteristic path length can serve as imaging biomarkers to predict cognitive impairment in patients with BC receiving neoadjuvant chemotherapy (Zhou et al., 2022; Yang et al., 2023). Quantitative MRI parameters (quantitative susceptibility mapping (QSM) and T2 star relaxation time map (T2*)) are more commonly used in neurodegenerative diseases. Besides, they have demonstrated potential as sensitive biomarkers for detecting microstructural brain changes following cancer treatment. A study (Chen et al., 2018a) reported that although QSM revealed no short-term differences in cancer or chemotherapy, it demonstrated a correlation with cognitive function, suggesting its potential as a biomarker in larger, longer-term studies. However, quantitative parameters such as QSM and T2* have rarely been used as post-treatment imaging predictors for cognitive impairment in NPC. Moreover, no study has integrated these metrics within a fully unified analysis pipeline from a single acquisition.

Furthermore, MRI sequences used in the aforementioned studies typically produce only one type of contrast per scan, limiting the imaging information obtained. If multiple contrasts are required, multiple sequences and scans must be performed. This process requires a long scanning time, making the images susceptible to contamination from head motion. Moreover, processing these data requires careful co-registration of multiple images, which often results in poor quality. Therefore, novel MRI technologies are urgently required. These technologies should ideally enable rapid and efficient acquisition of brain images with varying contrasts in a short scanning time to identify brain alterations following ICT.

Recently, multi-contrast and multiparametric MRI have emerged as promising techniques, enabling the acquisition of multiple contrasts and quantitative parameters from a single scan (Tong et al., 2020; Zhou et al., 2022; Yang et al., 2023). This method has been used to diagnose and quantitatively assess numerous diseases (Haacke et al., 2020; Narayana et al., 2020), including Parkinson’s disease and multiple sclerosis. However, existing multiparametric methods often exhibit limitations in terms of scan efficiency, image quality, and acquisition flexibility. Conversely, Multiplex (MTP) MRI has emerged as a rapidly evolving technique that addresses these challenges (Ye et al., 2022). Because of its flexible sequence design, MTP MRI enables integrated analysis of diverse imaging data, significantly reduces scanning time, and simplifies data processing, thereby ensuring high quantitative accuracy and reliability. To evaluate the ability of MTP to detect brain alterations following ICT more effectively than conventional approaches, we addressed a critical gap: no studies have used MTP as an imaging index for the effects of ICT on brain function and cognition. This methodological limitation suggests that MTP may provide deeper neurobiological insights into CRCI. If validated, MTP-derived biomarkers could potentially enable early clinical detection and intervention for CRCI in patients with NPC. Consequently, further research is essential to thoroughly investigate its potential application in assessing abnormalities in brain cognitive function following ICT in patients with NPC.

We hypothesized that patients with NPC would demonstrate significantly altered MTP parameters following ICT. Alterations in brain regions may correlate with potential cognitive changes after ICT, and altered parameters of these regions can predict cognitive decline.

## Materials and Methods

### Participants

**Figure 1:**
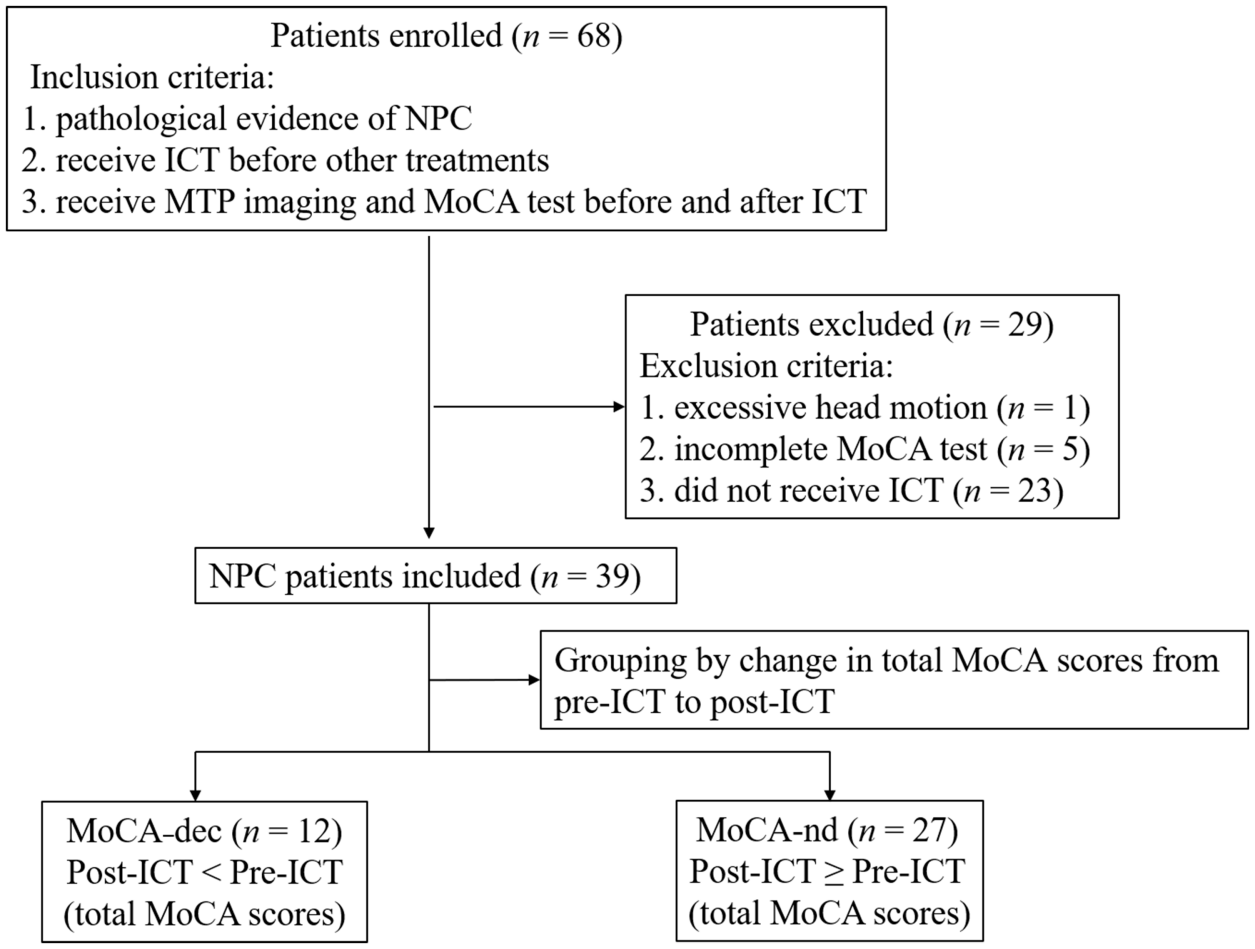
Flow diagram of patient selection for NPC. ICT, induction chemotherapy.

This study was approved by the Institutional Review Board of the Hubei Cancer Hospital (Approval No. LLHBCH2023YN-019, Date: April 10, 2023). This study was conducted in accordance with the Declaration of Helsinki. Written informed consent was obtained from all participants for the publication of this study. The inclusion criteria were as follows: (1) pathological evidence of NPC (stages III–IV at diagnosis according to the 8th edition of the Union for International Cancer Control (UICC)/American Joint Committee on Cancer (AJCC) staging system for NPC); (2) right-handed; (3) prepared to receive ICT before other treatments; (4) readiness to undergo the Montreal Cognitive Assessment (MoCA) before and after ICT. This study included 68 patients who were newly diagnosed with NPC between March 2023 and December 2023. After excluding 29 patients (two for missing MoCA assessments before ICT, one for excessive head motion, 23 for not undergoing ICT, and three for lacking MoCA evaluations after ICT), the final cohort comprised 39 patients. The selection process is illustrated in Fig. 1. These patients underwent MTP imaging before (pre-ICT) and after (post-ICT) receiving ICT. To explore the possible further influence of ICT on cognition, the patients were divided into two subgroups based on the change in their total MoCA scores: the MoCA-decreased group (MoCA-dec, *n* = 12), which consisted of patients who exhibited any reduction in total MoCA scores (post-ICT < pre-ICT), and the MoCA-non-decreased group (MoCA-nd, *n* = 27), which consisted of patients with stable or improved scores (post-ICT ≥ pre-ICT). This threshold was adopted to ensure the inclusion of all patients exhibiting potential cognitive decline, given the absence of a standardized MoCA threshold for ICT-related cognitive decline in this population.

The exclusion criteria were as follows: (1) brain invasion; (2) brain tumor or atrophy, neural-associated diseases, or a history of brain surgery; (3) any current medications that may affect cognitive function; or (4) inability to attend MRI scanning.

### MoCA tests

MoCA (Chen et al., 2016) (Chinese version) tests were performed on the same day as MRI scans to measure general cognitive performance in pre- and post-ICT groups. Patients completed the test within one week before starting the treatment for the pre-ICT baseline and again approximately three weeks (±2 days) after their final ICT cycle for the post-ICT follow-up. The authors have obtained permission to use MoCA from the MoCA Foundation. The MoCA test comprises seven cognitive domains: visuospatial and executive, naming, attention, language, abstraction, memory, and orientation. The total scores ranged from 0 to 30, and scores <26 indicated cognitive impairment.

### Treatments

A total of 39 patients received ICT, with 31 and eight patients undergoing ICT for two and three cycles, respectively. The salient characteristics of the study participants are presented in Table 1. All patients received ICT every three weeks with one of the following regimens: docetaxel plus cisplatin (DP), docetaxel plus carboplatin (DC), paclitaxel plus cisplatin (TP), or gemcitabine plus cisplatin (GP). These regimens were assigned by a multidisciplinary team following staging and in accordance with LANPC guidelines. Patients in the DP group were intravenously administered 75 mg/m^2^ docetaxel over 90 min on day 1 and 25 mg/m^2^ cisplatin daily on days 1–3 of the treatment. Patients in the DC group were intravenously administered 75 mg/m^2^ docetaxel on day 1 and carboplatin at a target area under the curve of 4–5 on day 1. A total of 135 mg/m^2^ paclitaxel was administered intravenously to patients in the TP group on day 1 and 25 mg/m^2^ cisplatin daily on days 1–3. The following regimen was administered to patients in the GP group as induction therapy: Intravenous administration of gemcitabine (1,000 mg/m^2^) on days 1 and 8 and cisplatin (25 mg/m^2^ daily) on days 1–3. Of the 39 patients, 17 received ICT in combination with programmed cell death protein 1 (PD-1) inhibitors.

**Table 1:** Basic characteristics of 39 patients.

Characteristic	Total (*n* = 39)	MoCA-dec (*n* = 12)	MoCA-nd (*n* = 27)	*P* value
Sex				
Male	25 (64.1%)	6 (50.0%)	19 (70.37%)	0.287
Female	14 (35.9%)	6 (50.0%)	8 (29.63%)	
Age (years)^*^	54.85 ± 8.78	54.50 ± 10.19	55.00 ± 8.28	0.872
Education (years)	9.51 ± 3.37	10.42 ± 3.29	9.11 ± 3.39	0.408
MoCA (pre-ICT)	26.21 ± 2.89	27.75 ± 2.70	25.52 ± 2.75	0.013
MoCA (post-ICT)	26.59 ± 2.79	25.67 ± 2.93	27.00 ± 2.67	0.178
T-classification (T1/T2/T3/T4)	3/11/21/4	1/4/6/1	2/7/15/3	NA
N-classification (N1/N2/N3)	6/19/14	2/6/4	4/13/10	NA
M-classification (M0/M1)	36/3	11/1	25/2	0.539
UICC/AJCC stage				
III	23 (59.0%)	8 (66.67%)	15 (55.56%)	0.726
IV	16 (41.0%)	4 (33.33%)	12 (44.44%)	
ICT (2-cycle/3-cycle)	33/6	9/3	24/3	0.645
ICT (DC/DP/TP/GP) + PD-1inhibitor	2/5/5/5	2/2/1/1	0/3/4/4	NA
ICT alone (DC/DP/TP/GP)	1/4/6/11	0/2/3/1	1/2/3/10	NA

**Notes.**

* Data are presented as mean ± standard deviation. *P* values were calculated using independent-samples *t* tests or Mann–Whitney U tests for continuous variables and Fisher’s exact tests for categorical variables. Fisher’s exact tests were applied because of the small expected cell counts.

UICC/AJCC Union for International Cancer Control/American Joint Committee on Cancer ICT induction chemotherapy MoCA Montreal Cognitive Assessment pre-ICT before induction chemotherapy post-ICT after induction chemotherapy

All patients were right-handed. NA, not applicable because of the large number of categories with small sample sizes.

### MRI acquisitions

All MRI data were acquired using a 3.0 T MRI scanner (uMRI 790; United Imaging Healthcare, Shanghai, China) with a 32-channel phased-array head coil. During MRI acquisition, each participant was positioned supine, with the head in a neutral position and comfortably fixed using a belt and foam pads. All participants were required to relax, avoid focused thoughts, stay awake, and keep their eyes closed. We collected a single MTP scan for each subject before and after ICT, which enabled the acquisition of multiple contrasts. These include augmented T1-weighted image, susceptibility weighted image, T1-weighted image, T2*, T1 relaxation time map (T1Map), proton density map (PDMap), R2 star relaxation rate map (R2*), and QSM. MTP data were collected using an MTP imaging sequence with the following parameters: repetition time = 35 ms, echo time =3.01/7.02/9.68/13.69/16.35/20.36/23.02 ms, voxel = 1  × 1  × 2 mm^3^, slice thickness/intersection gap = 1/0 mm, field of view = 224  × 224 mm^2^, matrix size = 224  × 224, and scan time = 6.21 min.

### Data preprocessing

MTP data were analyzed using the BrainTool (Wu et al., 2023) software (uMRI 790, United Imaging Healthcare, Shanghai, China). Brain images were precisely segmented into 106 bilateral brain regions using deep learning techniques. Subsequently, the segmented MTP images underwent normalization, multimodal fusion, and quantification analyses. Estimated total intracranial volume corrections were used for cortical structural analysis. The resulting differences in quantitative parameters, including the mean brain volume, mean T2*, mean QSM, mean T1Map, mean PDMap, and mean R2*, were obtained for each of the 106 bilateral brain regions. Finally, the normality of the data from the brain regions was assessed using the Kolmogorov–Smirnov test in IBM SPSS Statistics (version 26.0; IBM Corp., Armonk, NY, USA). All measured data satisfied the assumptions of normality.

### Statistical analysis

Demographic and clinical characteristics were compared between the MoCA-dec and MoCA-nd subgroups (Table 1). Continuous variables were analyzed using independent-samples *t* tests or Mann–Whitney U tests, with normality assessed by the Shapiro–Wilk test. Categorical variables were compared using Fisher’s exact test when the expected cell frequencies were < 5. Statistical significance was set at *P* < 0.05. All analyses were performed using SPSS (version 26.0).

#### Longitudinal changes of brain region parameters

To explore the longitudinally altered quantitative parameters between the pre- and post-ICT assessments, we performed a paired-samples permutation test using the Permutation Analysis of Linear Models (PALM) tool, as implemented in Data Processing & Analysis of Brain Imaging (DPABI) software (version 7.0; http://rfmri.org/dpabi). The analysis employed a paired contrast design within the general linear model (GLM) framework, with subject-specific intercepts modeled as nuisance variables to account for inter-subject variability. For each of the 106 bilateral brain regions, the pre-ICT *versus* post-ICT contrast was tested using permutation-based inference (10,000 permutations). The *t*-statistic served as the test statistic within this non-parametric permutation framework. Multiple comparisons across regions were corrected using PALM’s built-in false discovery rate (FDR) method, with significance set at *P*_FDR_ < 0.05. Age, sex, and education were not included as covariates in the PALM models. In this within-subject design, each patient served as their own control, thereby accounting for time-invariant factors, such as sex and education. The potential confounding effect of age is potentially minimal for two reasons: first, the short interval between scans (mean 52.23 ± 12.51 days; range 39–92 days) precludes significant age-related changes. Second, the cohort exhibited a relatively concentrated age distribution, primarily within the 50–60-year range (Supplementary Material A for age distribution histograms). This interval remained significantly unchanged across the cohorts.

#### MoCA correlation analysis

To mitigate the effect of inter-subject variability (such as baseline individual differences in brain size) and prevent bias from varying scales, we used the relative rate of change rather than absolute values for correlation analysis. This approach effectively normalizes each patient to their own baseline, enabling us to focus on the magnitude of changes during ICT. Particularly, the brain region value difference ratio (BR-R) was defined as the ratio of the difference between post- and pre-ICT brain region values divided by the pre-ICT brain region values. The MoCA score difference ratio (MS-R) was the ratio of the difference between post- and pre-ICT MoCA scores divided by pre-ICT MoCA scores. Subsequently, multiple linear regression analyses were performed using SPSS (version 26.0) to investigate the correlation between longitudinal changes in mean regional values and MoCA scores. In particular, we assessed the associations between (1) BR-R and MS-R; (2) brain region parameters of pre-ICT and MS-R; and (3) brain region parameters of pre-ICT and MoCA scores of post-ICT. Before regression analysis, Pearson’s correlation was used to screen potential predictors. In the models, age, sex, and years of education were included as covariates to mitigate potential confounding effects. The assumptions for multiple linear regression were rigorously verified. Normality of residuals was confirmed through probability-probability plots, homoscedasticity was assessed through scatterplots of standardized residuals, and independence of observations was checked using the Durbin–Watson statistic. Multicollinearity was evaluated using the variance inflation factor (VIF), and all values were < 5.0, indicating no significant collinearity. Additionally, influential points identified by Cook’s distance were retained to preserve the statistical power of this small cohort (*n* = 39) and to represent the inherent clinical heterogeneity of ICT-related neurotoxicity. All regression analyses were corrected for multiple comparisons using the Benjamini–Hochberg FDR method, with a significance threshold of *P*_FDR_ < 0.05. Furthermore, to thoroughly evaluate cognitive decline, the correlations between the seven subscores of MoCA and brain region parameters in patients with NPC were similarly analyzed.

#### Statistical analysis of MoCA-dec and MoCA-nd groups

Based on the longitudinally altered brain regions between pre- and post-ICT assessments, the quantitative MRI parameters in those regions were compared between MoCA-dec and MoCA-nd groups using analysis of covariance (ANCOVA). This included the brain region parameters in pre-ICT, post-ICT, and BR-R conditions. Age, sex, years of education, and baseline MoCA scores were included as the covariates. The assumptions for ANCOVA, including the normality of residuals (Shapiro–Wilk test) and homogeneity of variance (Levene’s test), were verified for all models (*P* > 0.05). In addition, the homogeneity of regression slopes was confirmed by evaluating the interaction between groups and covariates. All analyses were performed using SPSS (version 26.0). All *P*-values were FDR-corrected for multiple comparisons, with an adjusted *P* < 0.05 considered statistically significant.

#### Prediction of impaired cognitive function

All predictive modeling and statistical analyses were performed using R software (version 4.5.2; R Core Team, 2025). We used multivariate logistic regression to identify predictors of MoCA decline. The variables, including sex, baseline MoCA score, and the altered brain region parameters, demonstrated significant between-group differences in the prior ANCOVA comparing MoCA-dec and MoCA-nd groups. A subset selection procedure assessed 112 unique variable combinations, each containing at least one regional quantitative parameter. All continuous variables were center-standardized before analysis. Model performance and stability were assessed *via* receiver operating characteristic (ROC) analysis, combined with repeated 5-fold cross-validation with 30 repetitions (150 total validation folds), using the caret package (version 7.0.1).

The optimal model was selected based on the highest pooled area under the curve (AUC) calculated from the aggregated out-of-fold predictions. Leave-one-out cross-validation with Firth’s bias-reduced estimation (brglm2 version 1.0.1) estimated the 0–1 prediction error and assessed model stability in this small sample. The pooled AUC was calculated from a single ROC curve fitted to all aggregated out-of-fold predictions. This approach was preferred over the arithmetic mean of fold-specific AUCs, which can be inflated by folds with quasi-complete separation, as the pooled AUC better reflects the model’s overall discrimination across the complete dataset.

The 95% confidence interval (CI) for the pooled AUC was calculated using the percentile bootstrap method applied to the pooled out-of-fold predictions (1,000 iterations; boot package version 1.3.32). The pooled predictions were first generated through repeated 5-fold cross-validation (30 repetitions × 5 folds = 150 test folds). Bootstrap samples were then drawn with replacement from these pooled predictions, stratified by the observed outcome to preserve the class distribution. For each bootstrap iteration, an AUC was computed using the pROC package (version 1.19.0.1), and the 95% CI was derived as the 2.5th and 97.5th percentiles of the 1,000 bootstrap AUC estimates. Model calibration was evaluated using bootstrap resampling with 1,000 iterations (rms package version 8.1.0) to generate optimism-corrected calibration curves, assessing the agreement between predicted probabilities and observed outcome frequencies across risk deciles.

## Results

### Participant characteristics

Table 1 summarizes the demographic and clinical characteristics of all study participants. MoCA-dec and MoCA-nd subgroups were comparable with respect to sex, age, education, M stage, UICC/AJCC stage, and ICT cycles (all *P* > 0.05). However, the baseline MoCA scores differed significantly (*P* = 0.024) and were included as covariates in ANCOVA models.

### Longitudinal changes of brain region parameters

**Figure 2:**
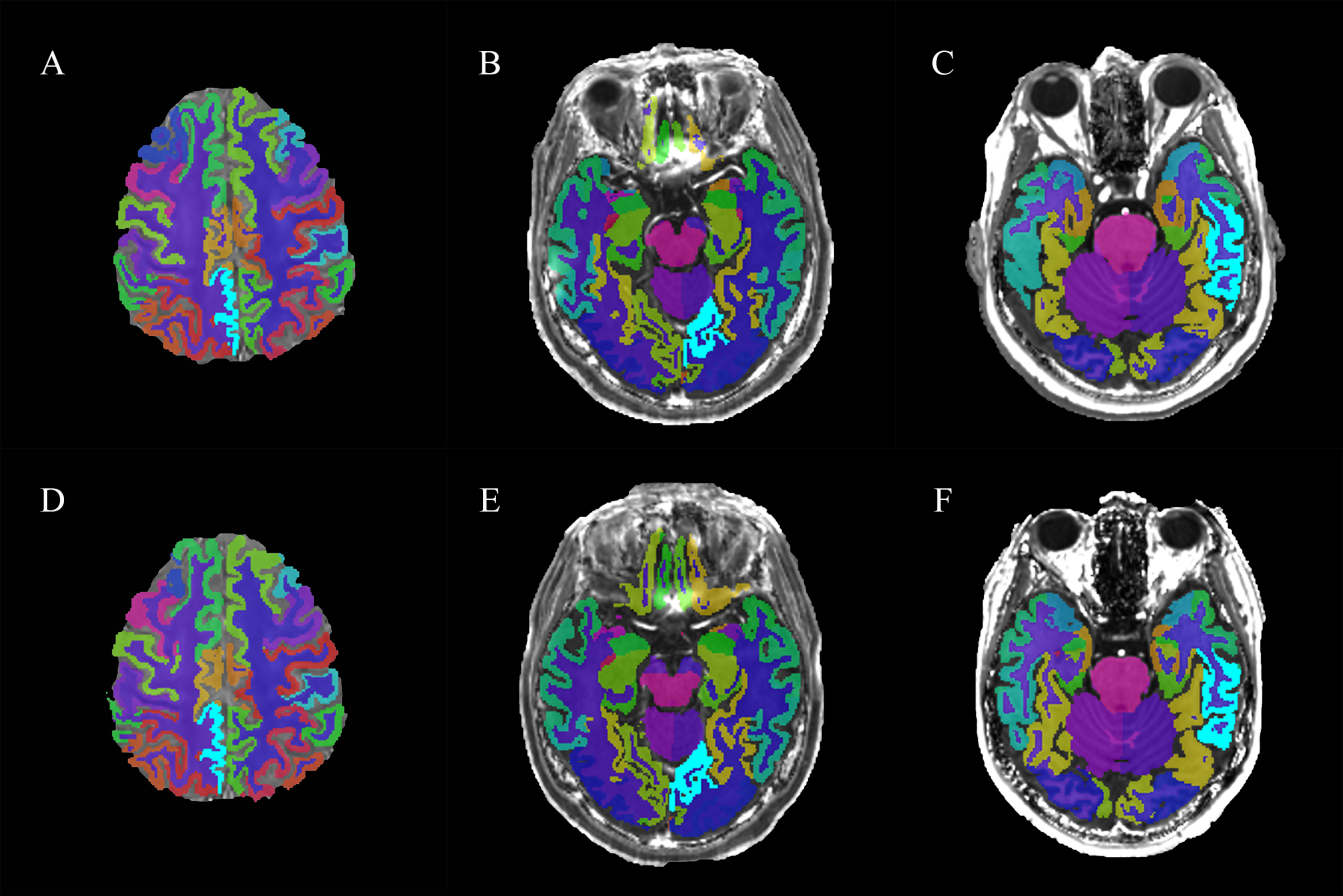
Visualization of longitudinal brain alterations in a patient with NPC before and after ICT. The blue-highlighted regions indicate specific longitudinal changes detected between pre- and post-ICT assessments. (A–C) Pre-ICT axial images displaying: (A) a quantitative susceptibility mapping (QSM) image of right precuneus, (B) an R2* map of left lingual gyrus, and (C) an anatomical aT1W image of left inferior temporal gyrus. (D–F) Post-ICT axial images from the same patient displaying: (D) a QSM image of right precuneus, (E) an R2* map of left lingual gyrus, and (F) an anatomical aT1W image of left inferior temporal gyrus. The brain was segmented into 106 bilateral regions using BrainTool software. These ROIs were overlaid on standard brain templates to visualize anatomical localization. The assigned colors were used solely to distinguish different anatomical structures and are arbitrary; they do not represent statistical values, signal intensities, or quantitative changes.

Longitudinal changes in brain region parameters between the pre- and post-ICT assessments are presented in Table 2. Not all brain region parameters showed significant changes in the longitudinal study. Four parameters were found to be significantly altered among these longitudinal changes, including QSM, T2*, R2*, and volume. Figure 2 depicts the altered brain regions in a patient with NPC at pre- and post-ICT.

**Table 2:** Results of MTP-derived brain parameters in patients with NPC at pre- and post-ICT assessments.

Quantitative parameters	Brain regions	Pre-ICT^*^	Post-ICT^*^	Average of paired differences	Permutation *t*	*P*_FDR_
QSM mean values (ppb)						
R precuneus, PCUN.R	Parietal lobe	4.669 ± 2.815	5.931 ± 2.323	1.262 ± 2.241	−3.516	0.023
R2* mean values						
R superior frontal gyrus, SFG.R	Frontal lobe	14.300 ± 0.607	14.005 ± 0.603	−0.295 ± 0.473	3.889	0.020
L paracentral lobule, PCL.L	Parietal lobe	15.828 ± 1.288	15.379 ± 1.168	−0.449 ± 0.881	3.182	0.040
L precuneus, PCUN.L	Parietal lobe	15.444 ± 0.839	15.049 ± 0.846	−0.395 ± 0.762	3.235	0.034
R opercula	Parietal lobe	14.459 ± 0.672	14.051 ± 0.826	−0.408 ± 0.828	3.074	0.043
R superior parietal lobule, SPL.R	Parietal lobe	15.351 ± 0.631	15.000 ± 0.631	−0.351 ± 0.569	3.856	0.020
R inferior parietal lobule, IPL.R	Parietal lobe	15.510 ± 0.718	15.044 ± 0.770	−0.467 ± 0.877	3.324	0.034
R supramarginal gyrus, SMG.R	Parietal lobe	15.256 ± 0.738	14.828 ± 0.798	−0.428 ± 0.825	3.240	0.034
L cuneus, CUN.L	Occipital lobe	18.079 ± 1.399	17.344 ± 1.413	−0.736 ± 0.945	4.861	0.011
L lingual gyrus, LING.L	Occipital lobe	18.377 ± 1.199	17.649 ± 1.462	−0.728 ± 1.287	3.533	0.027
L lateral occipital gyrus	Occipital lobe	17.649 ± 0.900	17.200 ± 0.917	−0.449 ± 0.863	3.249	0.040
R lateral occipital gyrus	Occipital lobe	17.821 ± 0.946	17.000 ± 0.933	−0.821 ± 0.893	5.736	0.011
R hippocampus, HIP.R	Temporal lobe	14.026 ± 1.062	13.526 ± 0.899	−0.500 ± 1.052	2.969	0.043
L isthmus of the cingulate gyrus	Subcortical lobe	16.777 ± 0.882	16.282 ± 1.306	−0.495 ± 0.973	3.175	0.038
T2* mean values						
R precentral gyrus, PreCG.R	Frontal lobe	73.818 ± 7.549	76.982 ± 9.063	3.164 ± 3.982	−4.963	0.018
R superior frontal gyrus, SFG.R	Frontal lobe	82.405 ± 5.829	85.187 ± 6.291	2.782 ± 4.218	−4.119	0.021
R postcentral gyrus, PoCG.R	Parietal lobe	75.051 ± 5.796	78.838 ± 8.562	3.787 ± 5.876	−4.025	0.018
R opercula	Parietal lobe	83.708 ± 9.274	88.574 ± 11.537	4.867 ± 8.614	−3.528	0.032
R superior parietal lobule, SPL.R	Parietal lobe	77.064 ± 6.932	81.218 ± 9.171	4.154 ± 6.796	−3.817	0.027
R inferior parietal lobule, IPL.R	Parietal lobe	70.992 ± 4.918	74.038 ± 6.368	3.046 ± 5.534	−3.438	0.030
R supramarginal gyrus, SMG.R	Parietal lobe	75.803 ± 4.635	79.238 ± 6.660	3.436 ± 5.510	−3.894	0.023
L cuneus, CUN.L	Occipital lobe	63.597 ± 5.025	66.790 ± 6.636	3.192 ± 4.331	−4.603	0.021
R lateral occipital gyrus	Occipital lobe	61.559 ± 3.197	64.015 ± 3.032	2.456 ± 2.881	−5.324	0.018
Volume mean values (mm^3^)						
R anterior middle frontal gyrus, AMF.R	Frontal lobe	14,774.283 ± 2,021.826	14,276.464 ± 2,005.314	−497.819 ± 1,025.707	3.031	0.032
L opercula	Parietal lobe	4,303.462 ± 799.775	4,213.462 ± 806.507	−90.000 ± 178.409	3.150	0.040
L superior parietal lobule, SPL.L	Parietal lobe	12,457.282 ± 1,852.412	12,182.693 ± 1,835.834	−274.589 ± 513.400	3.340	0.027
L supramarginal gyrus, SMG.L	Parietal lobe	11,065.462 ± 1,648.279	10,735.001 ± 1,573.224	−330.461 ± 487.032	4.237	0.011
R inferior parietal lobule, IPL.R	Parietal lobe	14,357.231 ± 2,092.684	14,074.078 ± 2,064.831	−283.153 ± 579.077	3.054	0.043
R precuneus, PCUN.R	Parietal lobe	9,922.641 ± 1,324.739	9,587.411 ± 1,142.040	−335.231 ± 408.920	5.120	0.011
R supramarginal gyrus, SMG,R	Parietal lobe	9,536.205 ± 1,370.268	9,341.205 ± 1,197.664	−195.000 ± 395.490	3.079	0.034
R lingual gyrus, LING.R	Occipital lobe	7,140.898 ± 1,132.922	6,908.923 ± 1,196.753	−231.975 ± 414.574	3.494	0.029
L fusiform gyrus, FFG.L	Temporal lobe	9,214.718 ± 1,186.040	8,862.154 ± 1,147.262	−352.564 ± 727.408	3.027	0.043
L middle temporal gyrus, MTG.L	Temporal lobe	10,647.257 ± 1,533.294	10,302.001 ± 1,593.418	−345.256 ± 588.870	3.662	0.020
L inferior temporal gyrus, ITG.L	Temporal lobe	10,920.744 ± 1,768.973	10,313.718 ± 1,925.893	−607.026 ± 1,151.396	3.292	0.034
R hippocampus, HIP.R	Temporal lobe	4,473.308 ± 462.234	4,339.436 ± 501.880	−133.872 ± 253.496	3.298	0.032
R fusiform gyrus, FFG.R	Temporal lobe	8,728.128 ± 1,214.450	8,417.769 ± 1,259.059	−310.359 ± 608.564	3.185	0.034
R inferior temporal gyrus, ITG.R	Temporal lobe	10,103.616 ± 1,352.999	9,453.847 ± 1,654.266	−649.769 ± 1,279.830	3.171	0.034
L insula, INS.L	Subcortical lobe	7,454.897 ± 782.547	7,278.410 ± 703.725	−176.487 ± 253.466	4.348	0.011
R posterior cingulate gyrus, PCG.R	Subcortical lobe	3,321.564 ± 469.906	3,178.744 ± 383.613	−142.821 ± 234.585	3.802	0.024
R isthmus of cingulate gyrus	Subcortical lobe	2,484.128 ± 339.058	2,396.667 ± 353.092	−87.462 ± 169.040	3.231	0.027
R amygdala, AMYG.R	Subcortical lobe	1,801.000 ± 230.016	1,724.769 ± 266.498	−76.231 ± 156.085	3.050	0.035

**Notes.**

* Data are presented as mean ± standard deviation.

L left QSM quantitative susceptibility mapping R right pre-ICT before induction chemotherapy post-ICT after induction chemotherapy

Permutation *t* statistics were derived from paired-samples permutation tests using the permutation analysis of linear models (PALM, 10,000 permutations). *P*_FDR_ values were corrected for multiple comparisons using the false discovery rate method.

Patients exhibited decreased mean R2* and increased mean T2* parameters in widespread brain regions at post-ICT compared to pre-ICT. These alterations were primarily localized in the parietal and occipital lobes, including the left cuneus (CUN.L), right superior frontal gyrus (SFG.R), right opercula, right superior parietal lobule (SPL.R), right inferior parietal lobule (IPL.R), right supramarginal gyrus, and right lateral occipital gyrus. Furthermore, increased mean T2* parameters were observed in the right precentral gyrus and postcentral gyrus. The mean R2* values decreased in the left paracentral lobule, left isthmus of the cingulate gyrus, left lingual gyrus (LING.L), left precuneus (PCUN.L), left lateral occipital gyrus, and right hippocampus (HIP.R). Finally, a significant increase in the mean QSM value was observed in the right precuneus (PCUN.R; *P*_FDR_ = 0.023).

Patients exhibited decreased mean volume values in 18 brain regions at post-ICT compared to pre-ICT, 12 of which were primarily located in the parietal lobe (left opercula, left superior parietal lobule (SPL.L), IPL.R, PCUN.R, and bilateral supramarginal gyrus) and temporal lobe (bilateral fusiform gyrus, left middle temporal gyrus, bilateral inferior temporal gyrus, and HIP.R). In addition, volume reductions in four subcortical regions were observed in the left insula, right posterior cingulate gyrus, right isthmus of the cingulate gyrus, and right amygdala (AMYG.R). Moreover, the volumes of the right anterior middle frontal gyrus (AMF.R) and right lingual gyrus (LING.R) were found to decrease at post-ICT.

### MoCA correlation analysis

**Figure 3:**
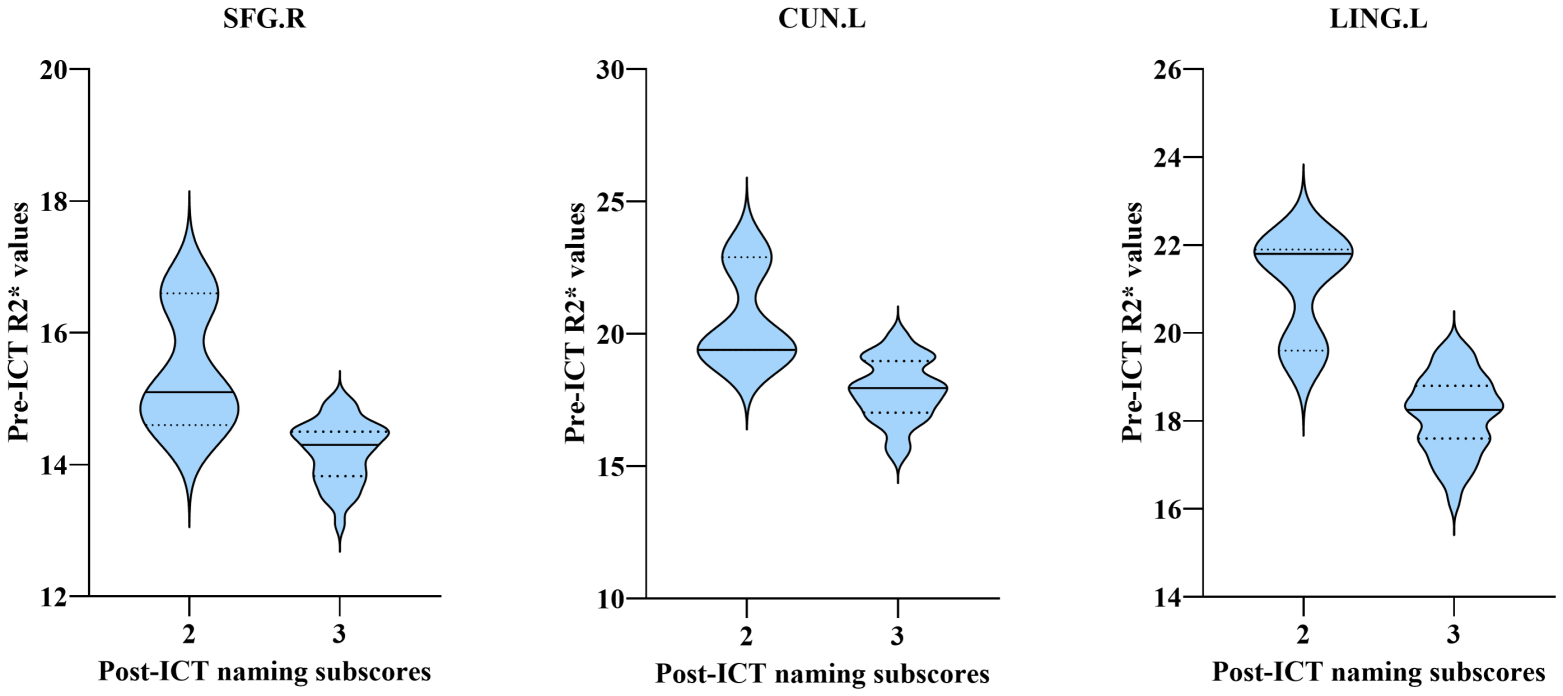
Pre-ICT R2* values in brain regions grouped according to post-ICT naming subscores. The violin plots illustrate the distribution of R2* values in patients with post-ICT naming subscores of 2 and 3. The solid line represents the median, and the dashed lines indicate the interquartile range. Baseline R2* values were significantly lower in patients with higher naming subscores (*P* < 0.001 for all comparisons). Specifically, elevated baseline R2* values in left cuneus (CUN.L) and left lingual gyrus (LING.L) (visual processing) and right superior frontal gyrus (SFG.R) (semantic retrieval) were correlated with poorer post-ICT naming performance. These findings suggest that pre-ICT R2* may serve as a potential imaging biomarker of individual vulnerability to naming decline in patients with NPC following ICT.

Although none of the correlations between longitudinal changes in mean regional values and MoCA total scores survived FDR correction (all *P*_FDR_ > 0.05), several uncorrected positive associations were observed. Notably, the change in the volume of the HIP.R and T2* parameters of the CUN.L in the BR-R showed a positive correlation with the MS-R (adjusted *R*^2^ = 0.015, *P* = 0.029, and adjusted *R*^2^ = 0.132, *P* = 0.002, respectively) (Supplementary Material B). After multiple linear regression analysis between the seven MoCA subscores and the quantitative MRI parameters, the pre-ICT R2* values in three regions remained significant after FDR correction and showed significant negative associations with post-ICT naming subscores (Table 3 and Fig. 3). The regression model for SFG.R was statistically significant (*F* (3, 34) = 4.401, *P* = 0.006), explaining 26.4% of the variance in naming subscores (adjusted *R*^2^ = 0.264). Baseline R2* of SFG.R showed a significant negative association with post-ICT naming subscores (*B* = −0.225, 95% CI [−0.354 to −0.096]; standardized *β* = −0.506, *t* = −3.545, *P* = 0.001). The model for CUN.L significantly predicted naming performance (*F* (3, 34) = 3.866, *P* = 0.011), with an adjusted *R*^2^ of 0.232. Baseline R2* of CUN.L showed a significant negative association with post-ICT naming subscores (*B* = −0.094, 95% CI [−0.152 to −0.035]; standardized *β* = −0.487, *t* = −3.262, *P* = 0.003). The LING.L model exhibited the highest explanatory power (*F* (3, 34) = 8.705, *P* < 0.001), with an adjusted *R*^2^ of 0.448. Baseline R2* of LING.L showed the strongest negative association among the three regions with post-ICT naming subscores (*B* = −0.152, 95% CI [−0.210 to −0.094]; standardized *β* = −0.673, *t* = −5.301, *P* < 0.001). No significant multicollinearity was observed (all VIF < 2.0). Additionally, demographic variables, including sex, age, and years of education, explained minimal additional variance in naming subscores (Δ*R*^2^ = 0.042–0.065).

**Table 3:** Multiple linear regression analysis predicting post-ICT MoCA naming subscores based on baseline R2* values (*n* = 39).

Predictor (Pre-ICT R2* values)	Model	*B* (SE)	*B* (95% CI)	*β*	*t*	*P*	*R* ^2^	Adjusted *R*^2^	Δ*R*^2^	*F*
SFG.R	M1	−0.243 (0.061)	−0.367, −0.119	−0.546	−3.969	<0.001	0.299	0.280	0.299	15.752 (1, 37)
	M2	−0.225 (0.064)	−0.354, −0.096	−0.506	−3.545	0.001	0.341	0.264	0.043	4.401 (3, 34)
CUN.L	M1	−0.100 (0.027)	−0.155, −0.045	−0.520	−3.701	0.001	0.270	0.250	0.270	13.695 (1, 37)
	M2	−0.094 (0.029)	−0.152, −0.035	−0.487	−3.262	0.003	0.313	0.232	0.042	3.866 (3, 34)
LING.L	M1	−0.150 (0.028)	−0.206, −0.094	−0.664	−5.406	<0.001	0.441	0.426	0.441	29.226 (1, 37)
	M2	−0.152 (0.029)	−0.210, −0.094	−0.673	−5.301	<0.001	0.506	0.448	0.065	8.705 (3, 34)

**Notes.**

ICT induction chemotherapy MoCA Montreal Cognitive Assessment SFG.R right superior frontal gyrus CUN.L left cuneus LING.L left lingual gyrus M1 unadjusted single-variable model M2 model adjusted for age, sex, and years of education adjusted *R*^2^ adjusted coefficient of determination *B* unstandardized beta coefficient *β* standardized beta coefficient Δ*R*^2^ change in *R*^2^ from M1 to M2

Baseline R2* values in the SFG.R, CUN.L, and LING.L were significantly associated with post-ICT naming subscores. The inclusion of demographic covariates in M2 explained minimal additional variance (Δ*R*^2^ = 0.042–0.065), with baseline R2* remaining the dominant predictor. All predictors remained significant after false discovery rate correction.

### Statistical analysis of MoCA-dec and MoCA-nd groups

After FDR correction, the MoCA-dec group exhibited significantly larger volumes in AMYG.R both before and after ICT, and smaller volumes in the left inferior temporal gyrus (ITG.L) after ICT compared with the MoCA-nd group (all *P*_FDR_ < 0.05). However, after adjusting for sex, age, education, and baseline MoCA, ANCOVA models (adjusted *R*^2^ = 0.464, 0.410, and 0.372, respectively) indicated that brain-volume variables did not produce a significant main effect of the MoCA group (*P* ≥ 0.162 for all models; Table 4). The effect of the MoCA group on AMYG.R volume was not significant either before ICT (*F* = 0.138, *P* = 0.713) or after ICT (*F* = 0.006, *P* = 0.941). Moreover, ITG.L failed to attain significance after ICT (*F* = 2.051, *P* = 0.162). Consistent with these findings, the effect sizes for the MoCA group were modest (partial *η*^2^ = 0.004, <0.001, and 0.059, respectively). However, sex demonstrated medium to large effects on these regional volumes (partial *η*^2^ = 0.448, 0.387, and 0.228, respectively).

**Table 4:** Analysis of covariance (ANCOVA) for effects of brain region volumes on MoCA-dec and MoCA-nd subgroups (*n* = 39).

Brain region	Source	SS	*df*	*MS*	*F*	*P*	Partial *η*^2^	Adjusted *R*^2^
AMYG.R in pre-ICT (Volumes mm^3^)	Model	1,075,340	5	215,068	7.590	<0.001	0.535	0.464
	Sex	760,299	1	760,299	26.830	<0.001	0.448	
	Age	94,255	1	94,255	3.326	0.077	0.092	
	Education	16,110	1	16,110	0.569	0.456	0.017	
	Baseline MoCA	1,473	1	1,473	0.052	0.821	0.002	
	MoCA group	3,897	1	3,897	0.138	0.713	0.004	
AMYG.R in post-ICT (Volumes mm^3^)	Model	1,316,302	5	263,260	6.284	<0.001	0.488	0.410
	Sex	874,273	1	874,273	20.869	<0.001	0.387	
	Age	95,680	1	95,680	2.284	0.140	0.065	
	Education	119,869	1	119,869	2.861	0.100	0.080	
	Baseline MoCA	24,100	1	24,100	0.575	0.454	0.017	
	MoCA group	233	1	233	0.006	0.941	<0.001	
ITG.L in post-ICT (Volumes mm^3^)	Model	64,030,442	5	12,806,088	5.494	<0.001	0.454	0.372
	Sex	22,717,359	1	22,717,359	9.747	0.004	0.228	
	Age	34,469,677	1	34,469,677	14.789	0.001	0.309	
	Education	411,100	1	411,100	0.176	0.677	0.005	
	Baseline MoCA	421,923	1	421,923	0.181	0.673	0.005	
	MoCA group	4,779,316	1	4,779,316	2.051	0.162	0.059	

**Notes.**

AMYG amygdala ITG inferior temporal gyrus R right L left SS type III sum of squares *df* degrees of freedom *MS* mean square partial *η*^2^ partial eta squared adjusted *R*^2^ adjusted coefficient of determination

The table presents an analysis of covariance results, with brain-region volume as the dependent variable and MoCA cognitive-decline grouping as the independent variable, after adjusting for sex, age, education, and baseline MoCA. Brain-volume variables did not yield a significant main effect of MoCA group (all *P* > 0.05). The smallest *P* value was observed for ITG.L (*P* = 0.162), indicating that pre- or post-ICT volumes of the AMYG.R and ITG.L were not independently associated with cognitive-decline group membership in this cohort.

### Prediction of impaired cognitive function

**Figure 4:**
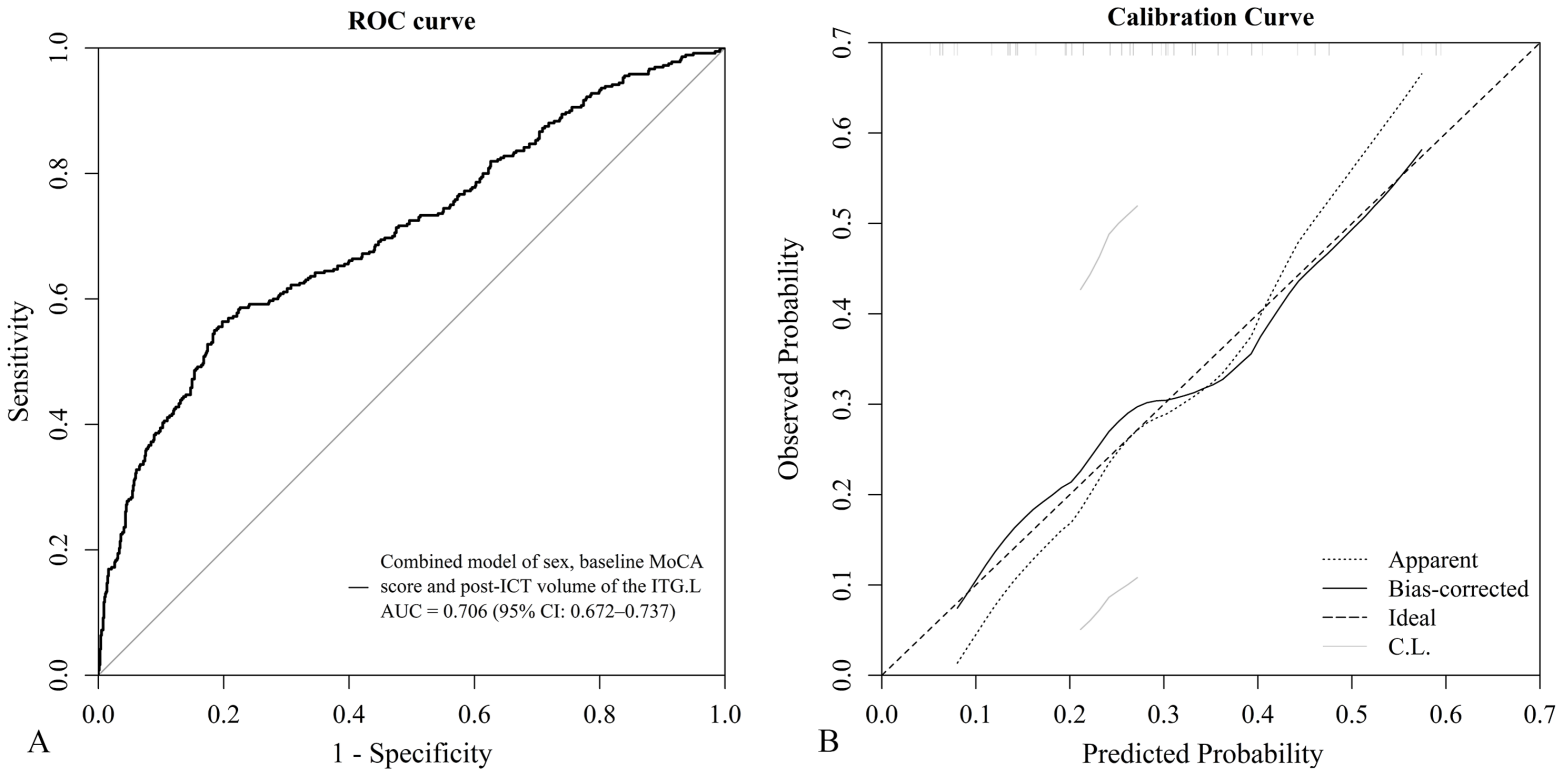
Evaluation of model performance. (A) The receiver operating characteristic (ROC) curve. The ROC was constructed from pooled out-of-fold predictions across 30 repetitions of 5-fold cross-validation (150 total validation folds). The area under the curve (AUC) was 0.706 (95% CI [0.672–0.737]), reflecting the discriminative ability of the combined model (sex, baseline MoCA score, and post-ICT volume of the left inferior temporal gyrus (ITG.L)) to distinguish between MoCA-decreased and MoCA-non-decreased groups. (B) Bootstrap optimism-corrected calibration curve (*B* = 1,000). The solid line represents the bias-corrected calibration, the dashed line represents the ideal perfect calibration, the dotted line represents the apparent calibration, and the gray lines indicate confidence limits (C.L.). The close agreement between apparent and bias-corrected curves suggests acceptable model calibration with limited evidence of overfitting. Mean absolute calibration error = 0.017.

The ROC analysis revealed that the optimal model included sex, baseline MoCA score, and post-ICT volume of the ITG.L. The optimal model achieved a pooled AUC of 0.706 (95% CI [0.672–0.737]) (Fig. 4A). Given the relatively small sample size, this model should be considered as preliminary and exploratory. The model demonstrated a sensitivity of 56.4% and a specificity of 80.2% at a threshold of 0.401. Bootstrap calibration produced a mean absolute error of 0.017, indicating good agreement between predicted and actual probabilities (Fig. 4B).

## Discussion

The MTP sequence represents an innovative approach for evaluating potential brain alterations (Gao et al., 2024). However, its application in evaluating microstructural and functional brain alterations in patients with NPC following ICT has not yet been reported. This study is the first to use the MTP sequence for functional assessment of ICT-related cognitive impairment. The following key findings were identified: First, widespread brain alterations were observed in patients with NPC at post-ICT compared to pre-ICT. These changes were primarily characterized by reduced brain volume and R2* in the DMN, and increased T2* parameters. Second, baseline R2* in the SFG.R and visual regions (CUN.L/LING.L) were significantly negatively associated with post-ICT domain-specific naming performance. Third, MoCA-dec and MoCA-nd groups exhibited significant differences in the quantitative brain region parameters. The combined model based on brain parameters and clinical variables achieved a preliminary and exploratory AUC for predicting cognitive impairment. Together, these findings offer preliminary evidence that MTP-derived parameters have the potential to serve as imaging biomarkers for detecting brain alterations and cognitive changes following ICT in patients with NPC.

### Widespread longitudinal brain alterations and acute-phase-related changes

In this prospective longitudinal study, we observed widespread brain changes in patients with NPC during the ICT period. These changes are characterized by decreased volume and R2*, and increased T2* and QSM across numerous regions, including the parietal, temporal, occipital, and frontal lobes. Our within-subject design effectively identified these time-related changes while minimizing inter-individual variability (Wu et al., 2025; Zhang et al., 2019b; Yang et al., 2023; Voon, Manan & Yahya, 2024). However, the lack of a concurrent healthy control group rendered it challenging to correlate these alterations solely with ICT-induced neurotoxicity. Tumor-related neuroinflammation and other factors may also be involved. Notwithstanding this limitation, these extensive changes suggest ICT-related brain disorganization in NPC. These alterations may reflect myelin damage, cortical atrophy, and decreased white matter integrity (Matsos et al., 2017; Niu et al., 2021; Dietrich, 2010; Pomykala et al., 2013), potentially impairing the brain’s ability to support neuronal adaptation and efficient information integration and segregation. This is consistent with the evidence from BC, where chemotherapy has been associated with disrupted white matter integrity (Matsos et al., 2017; Tong et al., 2020; Bai et al., 2021) and altered brain network efficiency (Yang et al., 2023).

Our results demonstrated that the parietal and subcortical lobes decreased in volume, with a high overlap with the DMN (Greicius et al., 2003). Given that the DMN is highly sensitive to physiological stressors, including systemic inflammation and oxidative stress, such changes may indicate treatment-related microstructural disruption. These stressors are often exacerbated during cancer treatment, leading to DMN disruption. Consistent with this interpretation, previous studies have linked DMN disruption to cognitive impairment following chemotherapy. Structural MRI demonstrated volume loss in the DMN in patients with NPC after CCRT (Zhang et al., 2019b). Rs-fMRI showed decreased ReHo in the DMN of patients with non-small cell lung cancer (Liu et al., 2022). Multivariate pattern analysis indicated that DMN functional connectivity could distinguish chemotherapy-treated patients with BC from controls (Kesler et al., 2013). Our findings extend this research by offering quantitative structural evidence of DMN involvement in patients with NPC following ICT, suggesting ICT-related alterations in cognitive function.

MRI enables non-invasive quantitative measurements of QSM, T2*, and R2*, offering unique insights into brain microstructural information. QSM maps the magnetic susceptibility of biological tissues determined by substances such as iron, hemosiderin, and myelin (Ghassaban et al., 2019; Harada et al., 2022; van der Weijden et al., 2023). It is particularly suited for monitoring relatively long-term iron accumulation (Zhang et al., 2019a). Although elevated QSM in regions such as the precuneus and HIP has been significantly correlated with cognitive decline in patients with chronic neurodegenerative diseases (Kim et al., 2017; Ayton et al., 2017), we observed only subtle QSM changes three weeks after ICT. This temporal mismatch suggests that QSM-detectable iron accumulation represents a chronic, cumulative process that may not be apparent within a short post-ICT period. Consistent with these findings, our increased QSM in the precuneus may suggest a trend toward elevated brain iron content and myelin loss in patients with NPC following ICT.

Notably, our results demonstrated more extensive alterations in T2* and R2* compared to QSM, particularly in the parietal lobe (primarily in IPL.R and PCUN.L) and the occipital lobe (mainly in the CUN.L and lateral occipital gyrus). In MRI physics, R2* is the reciprocal of T2* (R2* = 1/T2*) (Ji et al., 2025; Daugherty & Raz, 2015) . At the post-ICT assessment, we observed a significant increase in T2* and a corresponding decrease in R2*. Although T2* is traditionally sensitive to local magnetic field inhomogeneities (Ghassaban et al., 2019), such as iron deposition, it is also inherently affected by the underlying T2 relaxation time of the tissue (Ji et al., 2025; Daugherty & Raz, 2015). The predominant increase in T2* (and corresponding decrease in R2*) observed in the context of ICT-related neurotoxicity likely reflects a dominant contribution from elevated free water content, associated with neuroinflammation or extracellular edema. This effect may outweigh T2*-shortening effects expected from potential iron accumulation. This view is supported by our finding that T2* alterations were more widespread than QSM changes shortly after ICT. The widespread T2* elevation in DMN and visual processing regions (occipital lobe) may represent an acute-phase inflammatory response and microstructural water-content edema (Wefel & Schagen, 2012; Mounier et al., 2020), which could indicate a transient disruption of local tissue architecture. Although the lack of a non-ICT control group limits our ability to identify the specific cause of this inflammation, the MTP sequence enabled us to identify these subtle microenvironmental changes that may be undetectable on conventional anatomical imaging.

Moreover, the distribution of our preliminary findings is crucial for understanding the clinical course of chemobrain. Unlike chronic neuronal loss or extensive iron overload, inflammatory edema and early microstructural instability may represent acute-phase injuries that could potentially recover as the toxic effects of chemotherapy resolve (Yang et al., 2023). We hypothesize that these acute microstructural changes may be transient and reversible. However, as this study is based on a single three-week post-ICT time point, we cannot determine whether these alterations will resolve, persist, or progress to permanent injury. By combining QSM, T2*, and R2*, the MTP sequence enables the visualization of these subtle alterations, providing a methodological basis for characterizing early imaging changes. These findings generate hypotheses for future studies on the temporal evolution of such imaging alterations following ICT in patients with NPC.

### Baseline R2* as an indicator of vulnerability in the naming circuit

Our preliminary analysis suggests that baseline R2* values in the SFG.R, CUN.L, and LING.L were significantly negatively associated with post-ICT naming subscores. These regions support specialized functions in the cognitive process of naming, which transitions from visual perception to verbal expression. The cuneus and lingual gyrus are essential for complex visual processing and object recognition (Hahn, Ross & Stein, 2006; Gracitelli et al., 2020), whereas SFG is central for executive control and semantic retrieval (Zhang et al., 2025; Guo et al., 2025).

The lingual gyrus model accounted for a significant portion of the variance in naming score decline (adjusted *R*^2^ = 0.448, *P* < 0.001), whereas SFG and cuneus models demonstrated moderate effect sizes (adjusted *R*^2^ ranging from 0.232 to 0.264). The strength of these associations was comparable to that previously reported. For instance, Yang et al. (2023) reported *R*^2^ values of 0.19–0.30 between brain network alterations and clinical scores, while other key studies have reported correlation strengths equivalent to approximately *R*^2^ = 0.06 (Voon, Manan & Yahya, 2024) to *R*^2^ = 0.36 (Zhang et al., 2020b). The stronger association between the lingual gyrus and naming performance may be explained by its role in the visual-to-language pathway. As a critical component of the ventral visual pathway, it maps complex visual features to verbal names (Yablonski et al., 2024). Higher baseline R2* values in this region may reflect the pre-existing microstructural damage in the naming circuit. When subjected to the neurotoxic second hit of chemotherapy, this compromised node likely fails to maintain efficient information transfer, leading to specific language deficits (Yablonski et al., 2024). Consequently, baseline R2* of the lingual gyrus may merit further investigation as a potential marker of naming decline following ICT.

Although our findings are exploratory and may be influenced by the clinical heterogeneity of our cohort (for instance, tumor-node-metastasis (TNM) staging, regimens, and PD-1 inhibitors), the observed brain-behavior associations highlight the ability of MTP-derived parameters to detect ICT-related neural alterations in a real-world clinical population. Baseline R2* may therefore serve as a potential imaging biomarker of domain-specific brain-behavior associations in patients with NPC following ICT. Nevertheless, these findings remain hypothesis-generating and require validation in independent cohorts.

### Structural biomarkers and subgroup analysis of cognitive decline

Although the mean MoCA scores of the entire cohort did not decline significantly following ICT, this overall trend obscured considerable individual variation. Subgroup analysis (MoCA-dec *versus* MoCA-nd) revealed that structural alterations were more significant in patients experiencing cognitive decline. This subgroup-based approach appeared more sensitive for detecting ICT-related brain alterations than whole-group analysis.

Our longitudinal analysis suggested a general reduction in AMYG volume across the cohort, consistent with the known neurotoxic effects of chemotherapy on the limbic system (Ayton et al., 2017; Menning et al., 2017). Interestingly, we observed a paradoxical association. In the initial unadjusted comparisons, the MoCA-dec group had significantly larger AMYG volumes compared to the MoCA-nd group, a difference that was present both before and after ICT. This association between larger baseline regional volumes and poorer post-ICT cognitive outcomes may reflect a pre-existing state of brain vulnerability. However, after controlling for sex, age, education, and baseline MoCA, the effect of the MoCA group on AMYG.R volume was no longer statistically significant either before (*P* = 0.713) or after ICT (*P* = 0.941). The limited effect size for the MoCA group (partial *η*^2^ ≤ 0.001) compared to the substantial influence of sex (partial *η*^2^ ≥ 0.387) suggests that the initial inter-group differences were largely confounded by sex-related structural variability. Similarly, HIP also exhibited longitudinal volume loss, which may be associated with cognitive decline in episodic memory and spatial navigation (Comrie, Frank & Kay, 2022; Zhuang et al., 2023). However, its correlation with MoCA scores was no longer significant after FDR correction. Collectively, these findings suggest that limbic structures are susceptible to ICT (Ayton et al., 2017; Zhuang et al., 2023). However, their volumetric changes alone may be affected by demographic variables or lack sufficient sensitivity to predict global cognitive decline in this cohort.

Conversely, we observed a significant decrease in volume in the ITG following ICT, which was more pronounced in the MoCA-dec group. The temporal lobes are located within the irradiation field and are susceptible to severe damage following radiotherapy in patients with NPC (Yang et al., 2019; Kang et al., 2022; Zhang et al., 2022a; Zhang et al., 2019b; Zhang et al., 2020b). Previous investigations have revealed structural reductions in the ITG before radiotherapy (Zhang et al., 2019b; Zhang et al., 2020b). This supports our observation that ICT-related alterations may already be present before radiotherapy, which may be a key driver of early temporal lobe structural compromise.

However, our ANCOVA results indicated that ITG volume alone was insufficient to significantly distinguish between cognitive subgroups (*P* = 0.162), as individual variations in age and sex potentially masked the specific neurotoxic effects. When these demographic variables were combined, our predictive model (comprising sex, baseline MoCA, and post-ICT ITG.L volume) finally achieved an AUC of 0.706 (95% CI [0.672–0.737]). The improved model performance suggests that structural MRI features alone may be insufficient to explain the heterogeneity of cognitive outcomes following ICT. These findings suggest that integrating structural imaging with clinical variables may better capture inter-individual variability in ICT-related cognitive outcomes than single imaging measures. However, prediction models derived from small datasets may appear stronger than they actually are, particularly when evaluated on resampled data. Therefore, the model is preliminary and not ready for clinical use. Validation in larger, independent cohorts is required before any clinical application can be considered.

Finally, our findings should be interpreted in the context of the aforementioned clinical heterogeneity. This real-world diversity in treatment protocols introduces potential confounding effects that cannot be fully isolated due to the limited sample size. Accordingly, the observed longitudinal brain alterations and their associations with cognitive performance likely reflect the integrated neurobiological impact of these mixed treatment protocols, rather than a uniform ICT effect. Therefore, these biomarkers should be interpreted as indicators of ICT-related brain changes rather than specific markers of a single ICT regimen. Given the numerous brain regions, MRI parameters, and cognitive outcomes examined, our findings, with many comparisons and small samples, should be considered exploratory. Although FDR correction was applied, further validation in large-scale, homogeneous cohorts is required to confirm the stability of these findings and to clarify potential ICT-related neuroimaging patterns.

### Clinical feasibility of MTP

Our study suggests that the MTP sequence is a practical tool for monitoring ICT-related neurotoxic changes in the brain of patients with NPC. Compared with conventional MRI, MTP offers distinct practical advantages (Ye et al., 2022).

The primary advantage of MTP is its high efficiency. Conventional protocols typically require 15–25 min for separate acquisitions (Haacke et al., 2020), whereas MTP acquires all required data in a single 6.2-min scan. This 50%–70% reduction in scan time minimizes motion artifacts, which maintains image quality in patients with NPC who often require immobilization devices, thereby eliminating the need for sequence switching. Additionally, MTP provides inherently co-registered images. Conversely, conventional methods require 5–10 min for potentially suboptimal post-processing alignment. MTP eliminates the need for additional post-processing to align different sequences, thereby ensuring the accuracy and consistency of measurements within the brain microenvironment. These results can be obtained in < 1 min using the accompanying BrainTool software. Furthermore, MTP is designed for broad accessibility, relying on standard configurations present on modern 3T platforms (United Imaging, Siemens, GE, and Philips) and can be adopted through a simple software update.

Crucially, MTP-derived parameters, such as T2* and R2*, are sensitive to subtle microstructural and physiological alterations following ICT. Together with its technical advantages, MTP provides a time-efficient and integrated imaging framework that may facilitate the systematic investigation of ICT-related brain changes in patients with NPC. These measures should be considered exploratory imaging indices, enabling hypothesis generation regarding the temporal dynamics and regional vulnerability of brain tissue following ICT. These findings remain preliminary and require validation in prospective longitudinal studies with larger and more homogeneous cohorts.

### Limitations

This study has several limitations. First, the modest sample size (*n* = 39) and clinical heterogeneity (for instance, TNM staging, regimens, and PD-1 inhibitors) limit the statistical power and ability to perform stratified analyses. However, the longitudinal within-subject design effectively minimized inter-individual variability, thereby increasing the sensitivity of our findings. Second, the absence of a non-ICT control group hinders the definitive isolation of ICT-specific neurotoxicity from confounding factors, such as tumor-related systemic inflammation, paraneoplastic effects, cancer-related psychological stress, and natural aging. Consequently, our findings should be interpreted as ICT-related changes rather than definitive markers for a single regimen. Third, MoCA is a brief screening tool; our sensitivity threshold (any score reduction) may capture minor fluctuations unrelated to neurotoxicity. Future research should use comprehensive cognitive batteries and clinically validated threshold values. Finally, the single three-week post-ICT assessment precludes conclusions regarding whether these alterations are transient, persistent, or progressive. These results offer preliminary evidence of acute-phase changes and require validation in larger homogeneous cohorts with extended follow-up.

## Conclusions

In conclusion, this exploratory study suggests that the MTP sequence shows promise as an efficient and convenient approach for evaluating pre- and post-ICT changes in patients with NPC. Our study suggests that short-term ICT is associated with diminished brain volume and altered T2* and R2* values in DMN and visuospatial language regions. These MTP-derived multiparametric parameters provide preliminary evidence of their potential as emerging imaging biomarkers of ICT-related brain changes in patients with NPC. However, this study is limited to a single three-week post-ICT assessment, and it remains unclear whether these alterations are transient, persistent, or progressive. Therefore, the findings should be interpreted as preliminary evidence of acute-phase brain changes, and extended longitudinal follow-up is required to clarify their temporal evolution. Together, these findings generate hypotheses for future studies to evaluate imaging biomarkers of ICT-related brain changes and their potential relationship with cognitive outcomes in patients with NPC. This proposed framework can facilitate future studies with larger, controlled, and extended follow-up cohorts to further explore the neurological basis of cognitive impairment in patients with NPC following ICT.

##  Supplemental Information

10.7717/peerj.21650/supp-1 Supplemental Information 1 Age distribution histograms (*n* = 39) The age distribution of the 39 patients was relatively concentrated within the 50–60-year range.

10.7717/peerj.21650/supp-2 Supplemental Information 2 Multiple linear regression analysis predicting MS-R based on BR-R (*n* = 39) M1, unadjusted single-variable model; M2, model adjusted for age, sex, and years of education; adjusted *R*^2^, adjusted coefficient of determination; Δ *R*^2^, change in *R*^2^ from M1 to M2; F (*df*), incremental F-test; NA, not applicable. CUN.L, PCUN.R, and ITG.R were significant in M1 (*P* < 0.05), but Δ *R*^2^ after adding covariates was not significant (*P* ≥ 0.569), indicating that demographic variables did not enhance explanatory power. None of the predictors remained significant after false discovery rate (FDR) correction (*P*_FDR_ > 0.05).

10.7717/peerj.21650/supp-3 Supplemental Information 3 Raw data The clinical information and MoCA scores of the patients in NPC.

10.7717/peerj.21650/supp-4 Supplemental Information 4 MATLAB code for PALM permutation analysis of longitudinal brain changes This MATLAB code performs paired-samples permutation testing using the PALM tool (via DPABI) to investigate longitudinal changes in quantitative parameters between pre- and post-ICT assessments. The analysis utilizes a paired contrast design to identify significant differences across brain regions

10.7717/peerj.21650/supp-5 Supplemental Information 5 PALM design matrix for paired-samples permutation test This design matrix specifies the paired within-subject design for the PALM permutation analysis, with subject-specific intercepts modeled as nuisance variables to account for inter-subject variability. Used in conjunction with the contrast file (design1217-39.con) to test pre- versus post-induction chemotherapy differences.

10.7717/peerj.21650/supp-6 Supplemental Information 6 PALM contrast file for pre- versus post-ICT comparison This contrast file defines the paired comparison (pre- versus post-induction chemotherapy) for the PALM permutation analysis. Used in conjunction with the design matrix (design1217-39.mat) to identify significant longitudinal brain alterations.

10.7717/peerj.21650/supp-7 Supplemental Information 7 Dataset for ROC analysis predicting MoCA cognitive decline A raw data table containing the binary grouping variable (moca_bin), four covariates (age, sex, years of education, base_moca), and three brain-volume variables (right amygdala_pre, right amygdala_post, left inferior temporal gyrus_post) used for repeated 5-fold cross-validation ROC analyses.

10.7717/peerj.21650/supp-8 Supplemental Information 8 Codebook for ROC_data variables Variable definitions for ROC_data.csv, including binary MoCA outcome (0 = decline/MOCA-de), 1 = stable/MOCA-nd), demographic covariates (gender, age, education, baseline MoCA), and brain region volume predictors (roi_46, roi_47, roi_110) for logistic regression and ROC analyses.

10.7717/peerj.21650/supp-9 Supplemental Information 9 R code for selecting best AUC model combinations R script: reads ROC_data.csv, trains 112 glm models (each ≥ 1 brain ROI) via 10×5-fold CV, exports all AUCs to all_combinations_AUC-10.csv and reports the best formula.

10.7717/peerj.21650/supp-10 Supplemental Information 10 R code for cross-validated ROC analysis using pooled out-of-fold predictions with bootstrap confidence intervals Attachment Description: R Script for Predictive Modeling and Performance Evaluation Prediction Error Estimation: 1.Leave-one-out cross-validation using cv.glm to estimate the 0–1 prediction error of a Firth’s bias-reduced logistic regression (brglm2::brglmFit) predicting the MoCA binary outcome (decline/MoCA-de vs. stable/MoCA-). 2.Cross-Validation: Repeated 5-fold cross-validation (30 repetitions) using the caret pipeline to obtain cross-validated AUC, sensitivity, and specificity, with all predictors centered and scaled via preProcess. Predictions from all validation folds are aggregated for subsequent pooled AUC calculation. 3.Confidence Interval Estimation: Stratified percentile bootstrap (1,000 iterations) of pooled out-of-fold predictions using the boot package to derive the 95% confidence interval for the AUC, preserving class distribution via the strata argument. 4.ROC Analysis: ROC curve plotting and optimal cut-off identification (Youden index) with pROC, based on the pooled cross-validated predictions. 5.Calibration Assessment: Bootstrap optimism-corrected calibration curve (1,000 resamples) for the logistic model fitted with rms::lrm, with model calibration assessed via rms::calibrate.
Model Information: Formula: moca_bin gender + base_moca + roi_110 Software Versions: R v4.5.2, caret v7.0.1, pROC v1.19.0.1, boot v1.3.32, brglm2 v1.0.1, rms v8.1.0.

10.7717/peerj.21650/supp-11 Supplemental Information 11 STROBE checklist for the cohort study of pre- and post-induction chemotherapy brain changes and MoCA cognitive decline prediction

## Additional Information and Declarations

### Data Availability

The following information was supplied regarding data availability: The code and raw data are available in the Supplemental Files.
