# Supplementary material for: Brain alterations detected by multiplex magnetic resonance imaging in nasopharyngeal carcinoma patients after induction chemotherapy: a longitudinal study
Source: PeerJ. 2026 Aug 14;14:e21650. doi: 10.7717/peerj.21650 (PMC13480141; doi:10.7717/peerj.21650)
Supplement: Supplementary file 1 [file peerj-14-21650-s002.docx]

Supplementary Material B. Multiple linear regression analysis predicting MS-R based on BR-R (*n* = 39)

| Brain regions | Quantitative parameters | Model | *B* (SE) | *β* | *t* | P | *R*^2^ | Adjusted *R*^2^ | Δ*R*^2^ | *F* | *P* (F) |
| --- | --- | --- | --- | --- | --- | --- | --- | --- | --- | --- | --- |
| L opercula | Volume (mm³) | M1 | −0.631 (0.345) | −0.288 | −1.827 | 0.076 | 0.083 | 0.058 | NA | 3.337 (1,37) | 0.076 |
|  |  | M2 | −0.702 (0.371) | −0.320 | −1.891 | 0.067 | 0.116 | 0.012 | 0.033 | 0.427 (3,34) | 0.735 |
| R hippocampus, HIP.R | Volume (mm³) | M1 | 0.482 (0.245) | 0.307 | 1.965 | 0.057 | 0.094 | 0.070 | NA | 3.861 (1,37) | 0.057 |
|  |  | M2 | 0.485 (0.253) | 0.309 | 1.916 | 0.064 | 0.118 | 0.015 | 0.024 | 0.306 (3,34) | 0.821 |
| L cuneus, CUN.L | T2* | M1 | 0.645 (0.208) | 0.454 | 3.098 | 0.004 | 0.206 | 0.185 | NA | 9.598 (1,37) | 0.004 |
|  |  | M2 | 0.686 (0.231) | 0.483 | 2.965 | 0.005 | 0.224 | 0.132 | 0.018 | 0.260 (3,34) | 0.853 |
| R precuneus, PCUN.R | QSM(ppb) | M1 | 0.004 (0.002) | 0.342 | 2.216 | 0.033 | 0.117 | 0.093 | NA | 4.909 (1,37) | 0.033 |
|  |  | M2 | 0.005 (0.002) | 0.403 | 2.426 | 0.021 | 0.167 | 0.069 | 0.050 | 0.682 (3,34) | 0.569 |
| R inferior temporal gyrus, ITG.R | Volume (mm³) | M1 | 0.281 (0.120) | 0.359 | 2.341 | 0.025 | 0.129 | 0.106 | NA | 5.483 (1,37) | 0.025 |
|  |  | M2 | 0.279 (0.129) | 0.356 | 2.171 | 0.037 | 0.142 | 0.041 | 0.013 | 0.172 (3,34) | 0.915 |

M1, unadjusted single-variable model; M2, model adjusted for age, sex, and years of education; adjusted *R*², adjusted coefficient of determination; Δ*R*², change in *R*² from M1 to M2; F (*df*), incremental F-test; NA, not applicable.

CUN.L, PCUN.R, and ITG.R were significant in M1 (*P* < 0.05), but Δ*R*² after adding covariates was not significant (*P* ≥ 0.569), indicating that demographic variables did not enhance explanatory power. None of the predictors remained significant after false discovery rate (FDR) correction (*P*_FDR_ > 0.05).
