# Supplementary material for: Brain alterations detected by multiplex magnetic resonance imaging in nasopharyngeal carcinoma patients after induction chemotherapy: a longitudinal study
Source: PeerJ. 2026 Aug 14;14:e21650. doi: 10.7717/peerj.21650 (PMC13480141; doi:10.7717/peerj.21650)
Supplement: Supplementary file 2 [file peerj-14-21650-s011.docx]

STROBE Statement—checklist of items that should be included in reports of observational studies

|  | Item No. | Recommendation | Page  No. | Relevant text from manuscript |
| --- | --- | --- | --- | --- |
| **Title and abstract** | 1 | (*a*) Indicate the study’s design with a commonly used term in the title or the abstract | 1 | Title: "Brain alterations detected by multiplex magnetic resonance imaging in nasopharyngeal carcinoma patients after induction chemotherapy: a longitudinal study. |
|  |  | (*b*) Provide in the abstract an informative and balanced summary of what was done and what was found | 3–4 | **What was done:** "Thirty-nine patients underwent MTP imaging and MoCA before and after ICT. Paired-samples permutation test, multiple linear regression, ANCOVA, and multivariate logistic regression with ROC were performed." **What was found:** "Post-ICT patients exhibited decreased volume and R2*, increased T2* and QSM in DMN, occipital lobe, temporal lobe, and subcortical regions. Baseline R2* in SFG.R, CUN.L, and LING.L negatively predicted post-ICT naming scores. A combined model of sex, baseline MoCA score, and post-ICT ITG.L volume achieved an AUC of 0.706 for predicting cognitive decline. Findings are exploratory and lack a non-chemotherapy control." |
| Introduction | | | |  |
| Background/rationale | 2 | Explain the scientific background and rationale for the investigation being reported | 5–8 | Chemotherapy-related cognitive impairment (CRCI) is common in nasopharyngeal carcinoma (NPC) patients following induction chemotherapy (ICT), yet current studies are limited in their ability to visualize or quantify chemotherapy-induced brain changes. MRI-based indices have been established as neuroimaging biomarkers for evaluating post-treatment cognitive impairment in cancer patients. However, conventional MRI sequences typically produce only one type of contrast per scan, limiting the imaging information obtained. Multiplex (MTP) MRI enables simultaneous acquisition of multiple quantitative parameters (volume, R2*, T2*, QSM) in a single scan, providing a more comprehensive approach to characterize CRCI. This study aimed to evaluate MTP's potential for capturing chemotherapy-induced neurotoxicity more effectively than conventional approaches. |
| Objectives | 3 | State specific objectives, including any prespecified hypotheses | 8 | We hypothesized that patients with NPC would demonstrate significantly altered MTP parameters following ICT. Alterations in brain regions may correlate with potential cognitive changes after ICT, and altered parameters of these regions can predict cognitive decline. " |
| Methods | | | |  |
| Study design | 4 | Present key elements of study design early in the paper | 5-8 | Prospective longitudinal cohort study with pre- and post-ICT within-subject design |
| Setting | 5 | Describe the setting, locations, and relevant dates, including periods of recruitment, exposure, follow-up, and data collection | 1, 9–10 | Setting: Hubei Cancer Hospital, Tongji Medical College, Huazhong University of Science and Technology, Wuhan, China. Locations: Department of Radiology, Hubei Cancer Hospital. Dates: Recruitment: March 2023–December 2023; Exposure and follow-up: pre-ICT and 3 weeks (± 2 days) post-final ICT cycle; Data collection: MTP data and MoCA tests both performed same day as MRI scans. |
| Participants | 6 | (*a*) *Cohort study*—Give the eligibility criteria, and the sources and methods of selection of participants. Describe methods of follow-up  *Case-control study*—Give the eligibility criteria, and the sources and methods of case ascertainment and control selection. Give the rationale for the choice of cases and controls  *Cross-sectional study*—Give the eligibility criteria, and the sources and methods of selection of participants | 9–10 | Inclusion criteria: (1) pathological evidence of NPC (stages III-IV); (2) right-handed; (3) prepared to receive ICT before other treatments; (4) readiness to undergo MoCA before and after ICT. Exclusion criteria: (1) brain invasion; (2) brain tumor or atrophy; (3) medications affecting cognitive function; (4) inability to attend MRI.  Recruitment: 68 newly diagnosed NPC patients from March to December 2023 at Hubei Cancer Hospital. Of 68 patients screened, 39 were included after exclusions (Figure 1). Follow-up: pre-ICT and post-ICT assessments (mean 52.23 ± 12.51 days apart). Note: Participants subsequently categorized into MoCA-dec (*n* = 12) and MoCA-nd (*n* = 27) based on MoCA scores change for subgroup comparisons. |
|  |  | (*b*) *Cohort study*—For matched studies, give matching criteria and number of exposed and unexposed  *Case-control study*—For matched studies, give matching criteria and the number of controls per case | NA | Not applicable. No matching was performed in this cohort study. |
| Variables | 7 | Clearly define all outcomes, exposures, predictors, potential confounders, and effect modifiers. Give diagnostic criteria, if applicable | 10–12,19 | Exposure: ICT (DP/DC/TP/GP regimens; 17 with PD-1 inhibitors). Outcomes: MTP parameters (volume, T2*, QSM, T1Map, PDMap, R2*) and MoCA total/sub-scores. Predictors: baseline R2* (SFG.R, CUN.L, LING.L) and brain volumes (ITG.L, AMYG.R). Confounders: age, sex, education, baseline MoCA. Effect modifier: None formally tested; sex showed large main effects but was not evaluated as an effect modifier of group differences. Diagnostic criteria: NPC stage III–IV per UICC/AJCC 8th; cognitive impairment: MoCA < 26 |
| Data sources/ measurement | 8* | For each variable of interest, give sources of data and details of methods of assessment (measurement). Describe comparability of assessment methods if there is more than one group | 10–12 | All 39 patients received ICT (exposed group). No unexposed group exists in this single-arm cohort. MTP parameters: 3.0T MRI (uMRI 790) with a 32-channel head coil. Parameters: TR = 35 ms, TE = 3.01/7.02/9.68/13.69/16.35/20.36/23.02 ms, voxel = 1×1×2 mm³, scan time = 6.21 min. MoCA (Chinese version) was administered on the same day as MRI scans.; BrainTool software segmented 106 regions. MoCA: Chinese version, same day as MRI, MoCA Foundation permission. Demographics and clinical data: medical records and multidisciplinary team. Subgroup comparability: MoCA-dec and MoCA-nd groups used identical MRI and MoCA assessment methods. Longitudinal comparability: pre-ICT and post-ICT assessments used same protocols. |
| Bias | 9 | Describe any efforts to address potential sources of bias | 12–16, 29 | To address potential biases: (1) paired-samples permutation test with each patient as own control, adjusting for time-invariant confounders (sex, education); (2) relative change ratios (BR-R, MS-R) used instead of absolute values; (3) FDR correction for multiple comparisons (*P*_FDR_ < 0.05); (4) age, sex, education, baseline MoCA included as covariates in regression and ANCOVA; (5) repeated 5-fold cross-validation (30 repetitions) with Firth's bias-reduced estimation for prediction models; (6) automated BrainTool software to minimize assessment bias; (7) explicit inclusion/exclusion criteria with flow diagram (Figure 1); (8) lack of non-chemotherapy control group acknowledged as limitation. |
| Study size | 10 | Explain how the study size was arrived at | 9, 29 | No formal sample size calculation was performed given the exploratory nature of this study. Sample size was based on consecutive recruitment of eligible patients during the study period (March–December 2023). Of 68 newly diagnosed NPC patients, 29 were excluded (2 missing pre-ICT MoCA, 1 excessive head motion, 23 not undergoing ICT, 3 missing post-ICT MoCA). The final cohort comprised 39 patients who met eligibility criteria and completed both pre- and post-ICT assessments. The modest sample size (*n* = 39) is acknowledged as a limitation (Discussion, page 29) |

Continued on next page

| Quantitative variables | 11 | Explain how quantitative variables were handled in the analyses. If applicable, describe which groupings were chosen and why | 12, 14, 9 | Quantitative variables (MTP parameters, MoCA scores, age, education) were analyzed as continuous variables. Normality confirmed by Kolmogorov-Smirnov test (page 12). Relative change ratios were used: BR-R = (post-ICT − pre-ICT)/pre-ICT; MS-R = (post-ICT − pre-ICT)/pre-ICT. Grouping: MoCA-dec (post-ICT MoCA < pre-ICT, any decrease) vs MoCA-nd (post-ICT MoCA ≥ pre-ICT). Rationale: 'to ensure inclusion of all patients exhibiting potential cognitive decline, given the absence of a standardized MoCA threshold for chemotherapy-related cognitive decline in this population' (page 5) |
| --- | --- | --- | --- | --- |
| Statistical methods | 12 | (*a*) Describe all statistical methods, including those used to control for confounding | 12–16 | Paired-samples permutation test (PALM, 10,000 permutations) for longitudinal changes with FDR correction (*P*_FDR_ < 0.05). Multiple linear regression (adjusted for age, sex, education) for MoCA correlations. ANCOVA (adjusted for age, sex, education, baseline MoCA) for group comparisons. Multivariate logistic regression with ROC analysis for prediction. Repeated 5-fold CV (30 repetitions) and LOOCV with Firth's bias-reduced estimation for model validation. |
|  |  | (*b*) Describe any methods used to examine subgroups and interactions | 15, 19 | Subgroup analysis: MoCA-dec (post-ICT MoCA < pre-ICT) vs MoCA-nd (post-ICT MoCA ≥ pre-ICT). ANCOVA compared parameters between subgroups, controlling for age, sex, education, baseline MoCA. Interaction between group and covariates was tested for homogeneity of regression slopes. Sex showed large main effects (partial *η²* = 0.448, 0.387, and 0.228, respectively) but was not evaluated as an effect modifier of group differences. |
|  |  | (*c*) Explain how missing data were addressed | 9, Figure1 | Complete case analysis (*n* = 39). Exclusions: 29/68 excluded (2 missing pre-ICT MoCA, 1 excessive head motion, 23 not undergoing ICT, 3 missing post-ICT MoCA). No imputation performed. |
|  |  | (*d*) *Cohort study*—If applicable, explain how loss to follow-up was addressed  *Case-control study*—If applicable, explain how matching of cases and controls was addressed  *Cross-sectional study*—If applicable, describe analytical methods taking account of sampling strategy | 9 | No loss to follow-up. All 39 patients who completed pre-ICT assessments also completed post-ICT assessments. |
|  |  | (*e*) Describe any sensitivity analyses | 15–16 | (1) repeated 5-fold cross-validation (30 repetitions) with Firth's bias-reduced estimation to assess model stability in small sample; (2) bootstrap with 1,000 iterations for AUC 95% CI; (3) calibration penalty to address convergence; (4) pooled AUC preferred over mean AUC to avoid quasi-complete separation inflation |
| Results | | | | |
| Participants | 13* | (a) Report numbers of individuals at each stage of study—eg numbers potentially eligible, examined for eligibility, confirmed eligible, included in the study, completing follow-up, and analysed | 9, Figure 1 | Screened: 68. Excluded: 29 (2 missing pre-ICT MoCA, 1 head motion, 23 no ICT, 3 missing post-ICT MoCA). Included: 39. Completed follow-up: 39. Analysed: 39. |
|  |  | (b) Give reasons for non-participation at each stage | 9, Figure 1 | Exclusion reasons: missing pre-ICT MoCA (*n* = 2), excessive head motion (*n* = 1), did not undergo ICT (*n* = 23), missing post-ICT MoCA (*n* = 3). |
|  |  | (c) Consider use of a flow diagram | Figure 1 | Flow diagram provided (Figure 1) |
| Descriptive data | 14* | (a) Give characteristics of study participants (eg demographic, clinical, social) and information on exposures and potential confounders | 9–10, Table 1 | Participant characteristics (age, sex, education, clinical stage, ICT cycles, regimens, PD-1 use, baseline MoCA) presented in Table 1 for total cohort (*n* = 39) and subgroups (MoCA-dec, *n* = 12; MoCA-nd, *n* = 27). Exposures: ICT (DP/DC/TP/GP regimens). Confounders: age, sex, education, baseline MoCA. |
|  |  | (b) Indicate number of participants with missing data for each variable of interest | 9 | None in analyzed cohort (*n* = 39); missing data only in excluded patients (*n* = 29) |
|  |  | (c) *Cohort study*—Summarise follow-up time (eg, average and total amount) | 13 | Mean follow-up time between pre-ICT and post-ICT assessments: 52.23 ± 12.51 days (range 39–92 days) |
| Outcome data | 15* | *Cohort study*—Report numbers of outcome events or summary measures over time | 16–19, Tables 1–4, Figure 3 | Cognitive decline (MoCA-dec) occurred in 12/39 patients over the ICT period.  MoCA scores: baseline 25.95 ± 2.66 → post-ICT 25.51 ± 3.08.  Brain alterations: decreased volume (18 regions), decreased R2* (widespread), increased T2* (widespread), increased QSM (PCUN.R).  Predictors of naming decline: baseline R2* in SFG.R, CUN.L, LING.L (Table 3).  Group differences: AMYG.R volume larger in MoCA-dec; ITG.L volume smaller post-ICT in MoCA-dec (Table 4). |
|  |  | *Case-control study—*Report numbers in each exposure category, or summary measures of exposure | *NA* | *NA* |
|  |  | *Cross-sectional study—*Report numbers of outcome events or summary measures | *NA* | *NA* |
| Main results | 16 | (*a*) Give unadjusted estimates and, if applicable, confounder-adjusted estimates and their precision (eg, 95% confidence interval). Make clear which confounders were adjusted for and why they were included | 19–20, Tables 3–4, Figures 3–4 | Unadjusted: MoCA-dec vs MoCA-nd group differences in AMYG.R and ITG.L volumes (Section 3.4, Table 4). Adjusted (ANCOVA): After controlling for sex, age, education, and baseline MoCA, group effects on AMYG.R and ITG.L were non-significant (*P* ≥ 0.162, partial *η²* = 0.004–0.059). Regression (adjusted): Baseline R2* in SFG.R (*B* = −0.225, 95% CI [−0.354, −0.096], standardized *β* = −0.506, P = 0.001), CUN.L (*B* = −0.094, 95% CI [−0.152, −0.035], standardized *β* = −0.487, P = 0.003), and LING.L (*B* = −0.152, 95% CI [−0.210, −0.094], standardized *β* = −0.673, P < 0.001) negatively predicted naming scores, adjusted for age, sex, and education. Prediction model (adjusted): Combined model of sex, baseline MoCA, and post-ICT ITG.L volume: pooled AUC = 0.706 (95% CI: 0.672–0.737), sensitivity 56.4%, specificity 80.2% at threshold 0.401. |
|  |  | (*b*) Report category boundaries when continuous variables were categorized | 9–10 | MoCA-dec defined as post-ICT MoCA < pre-ICT (any decrease); MoCA-nd defined as post-ICT MoCA ≥ pre-ICT. Rationale: no standardized MoCA threshold for CRCI in NPC. No other continuous variables were categorized. |
|  |  | (*c*) If relevant, consider translating estimates of relative risk into absolute risk for a meaningful time period | NA | Not applicable (no relative risk estimates reported) |

Continued on next page

| Other analyses | 17 | Report other analyses done—eg analyses of subgroups and interactions, and sensitivity analyses | 14–16, 19–20, Table 4, Figure 4 | Subgroup analyses: MoCA-dec vs MoCA-nd compared via ANCOVA (controlling for age, sex, education, baseline MoCA). Interaction: homogeneity of regression slopes tested (*P* > 0.05); Sex had large main effects (partial *η²* = 0.228–0.448) but was not tested as effect modifier. Sensitivity analyses: repeated 5-fold cross-validation (30 repetitions), bootstrap with 1,000 iterations, Firth's bias-reduced estimation, calibration penalty. |
| --- | --- | --- | --- | --- |
| Discussion | | | | |
| Key results | 18 | Summarise key results with reference to study objectives | 20 | First, widespread brain alterations were observed in patients with NPC at post-ICT compared to pre-ICT. These changes were primarily characterized by reduced brain volume and R2* in the DMN, and increased T2* parameters. Second, baseline R2* in the SFG.R and visual regions (CUN.L/LING.L) were significant negative predictors of domain-specific naming after ICT. Third, MoCA-dec and MoCA-nd groups exhibited significant differences in the quantitative brain region parameters. The combined model based on brain parameters and clinical variables achieved a preliminary and exploratory AUC for predicting cognitive impairment. |
| Limitations | 19 | Discuss limitations of the study, taking into account sources of potential bias or imprecision. Discuss both direction and magnitude of any potential bias | 29–30 | (1) modest sample size (*n* = 39) and clinical heterogeneity (TNM, regimens, PD-1) limit statistical power and stratified analyses (potential false negatives). (2) No non-ICT control group—cannot isolate chemotherapy-specific neurotoxicity from tumor-related inflammation or aging (bias direction uncertain). (3) MoCA screening tool with 'any decrease' threshold may capture minor fluctuations (potential overestimation of decline). (4) Single post-ICT timepoint (3 weeks)—cannot determine if alterations are transient or progressive. (5) Mixed treatment protocols (DP/DC/TP/GP, ±PD-1)—regimen-specific effects cannot be isolated. |
| Interpretation | 20 | Give a cautious overall interpretation of results considering objectives, limitations, multiplicity of analyses, results from similar studies, and other relevant evidence | 20-30 | Objectives: MTP demonstrates potential for evaluating brain alterations in NPC following ICT; multiparametric indices provide preliminary evidence as emerging imaging biomarkers for early monitoring of chemotherapy-related cognitive impairment. Limitations: Findings should be interpreted with caution due to small sample (*n* = 39), lack of non-chemotherapy control, single three-week post-ICT assessment, and exploratory nature. Multiplicity: Numerous brain regions, MRI parameters, and cognitive outcomes examined; FDR correction applied but chance findings possible. Similar studies: Consistent with prior evidence of DMN disruption in cancer patients (NPC after CCRT, NSCLC, breast cancer), chemotherapy-related white matter/gray matter changes, and quantitative MRI biomarkers in neurodegenerative diseases. Relevant evidence: MTP's single-scan multi-parameter acquisition addresses conventional MRI limitations (motion artifacts, co-registration errors, scan time); clinical feasibility supported by 6.2-minute scan time and automated BrainTool processing. Cautious interpretation: These findings offer preliminary evidence of acute-phase microstructural changes and potential predictive value, but require validation in larger controlled cohorts with extended follow-up before clinical application. |
| Generalisability | 21 | Discuss the generalisability (external validity) of the study results | 9–11, 28–30 | Results generalisable to locally advanced NPC (stages III–IV) patients receiving ICT. MTP sequence is broadly accessible on modern 3T platforms (United Imaging, Siemens, GE, Philips) via software update (page 11). However, generalisability is limited by single-centre design, modest sample size (*n* = 39), absence of non-ICT control, and exploratory nature (page 12). Findings require validation in larger, more homogeneous, multi-centre cohorts before clinical application. |
| Other information | |  | | |
| Funding | 22 | Give the source of funding and the role of the funders for the present study and, if applicable, for the original study on which the present article is based | Not applicable (submitted separately) | This work was supported by the Hubei Provincial Key Technology Foundation of China [grant number 2021ACA013]; and the National key Research and Development Project of China [grant number 2018YFA0704000]. Yulin Liu was supported by the Chutian Scholars Program of Hubei Province. The funding agencies had no role in study design, data collection, analysis, interpretation, manuscript writing, or submission decision. |

*Give information separately for cases and controls in case-control studies and, if applicable, for exposed and unexposed groups in cohort and cross-sectional studies.

**Note:** An Explanation and Elaboration article discusses each checklist item and gives methodological background and published examples of transparent reporting. The STROBE checklist is best used in conjunction with this article (freely available on the Web sites of PLoS Medicine at http://www.plosmedicine.org/, Annals of Internal Medicine at http://www.annals.org/, and Epidemiology at http://www.epidem.com/). Information on the STROBE Initiative is available at www.strobe-statement.org.
